# Recent advances in photoelectrochemical platforms based on porous materials for environmental pollutant detection

**DOI:** 10.1039/d4ra00503a

**Published:** 2024-03-06

**Authors:** Shiben Liu, Jinhua Zhan, Bin Cai

**Affiliations:** a School of Chemistry and Chemical Engineering, Shandong University 250100 Jinan China bin.cai@sdu.edu.cn

## Abstract

Human health and ecology are seriously threatened by harmful environmental contaminants. It is essential to develop efficient and simple methods for their detection. Environmental pollutants can be detected using photoelectrochemical (PEC) detection technologies. The key ingredient in the PEC sensing system is the photoactive material. Due to the unique characteristics, such as a large surface area, enhanced exposure of active sites, and effective mass capture and diffusion, porous materials have been regarded as ideal sensing materials for the construction of PEC sensors. Extensive efforts have been devoted to the development and modification of PEC sensors based on porous materials. However, a review of the relationship between detection performance and the structure of porous materials is still lacking. In this work, we present an overview of PEC sensors based on porous materials. A number of typical porous materials are introduced separately, and their applications in PEC detection of different types of environmental pollutants are also discussed. More importantly, special attention has been paid to how the porous material's structure affects aspects like sensitivity, selectivity, and detection limits of the associated PEC sensor. In addition, future research perspectives in the area of PEC sensors based on porous materials are presented.

## Abstract 2

This review investigated the breakthroughs in porous photoactive materials such as metal oxides, metal–organic frameworks, covalent organic frameworks, graphitic carbon nitride, and MXene for the PEC detection of environmental contaminants.

## Graphical abstract

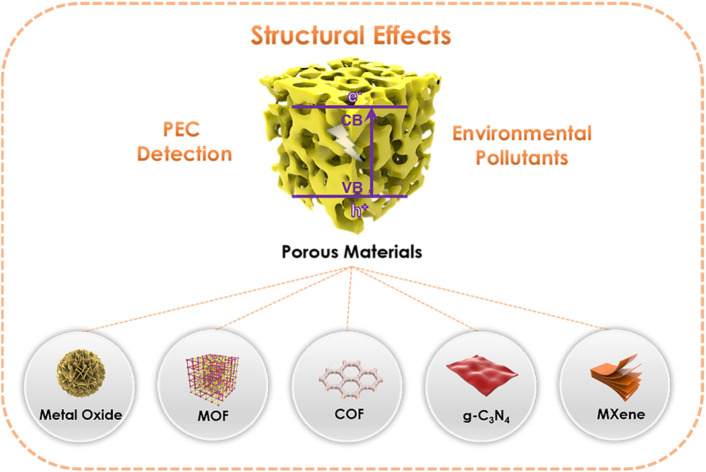

## 1. Introduction

Over the past few decades, global population growth and industrial development have resulted in serious environmental pollution.^1,2^ Substantial amounts of harmful chemical compounds, such as heavy metal ions, antibiotics, pesticides, and phenolics, have been discharged into the environment. The self-purification capacity of natural water bodies cannot cope with these increasing environmental pollutants, posing a severe threat to both the ecological environment and public health.^3,4^ These contaminants can be identified using traditional detection techniques like high-performance liquid chromatography, atomic fluorescence spectroscopy, atomic absorption/emission spectrometry, and inductively coupled plasma mass spectrometry.^5–9^ However, the aforementioned detection techniques are limited by the large experimental instruments, specific operating conditions, and high training costs.^10,11^ Therefore, it is crucial to develop efficient techniques for the sensitive identification of these environmental contaminants.

**Fig. 1:**
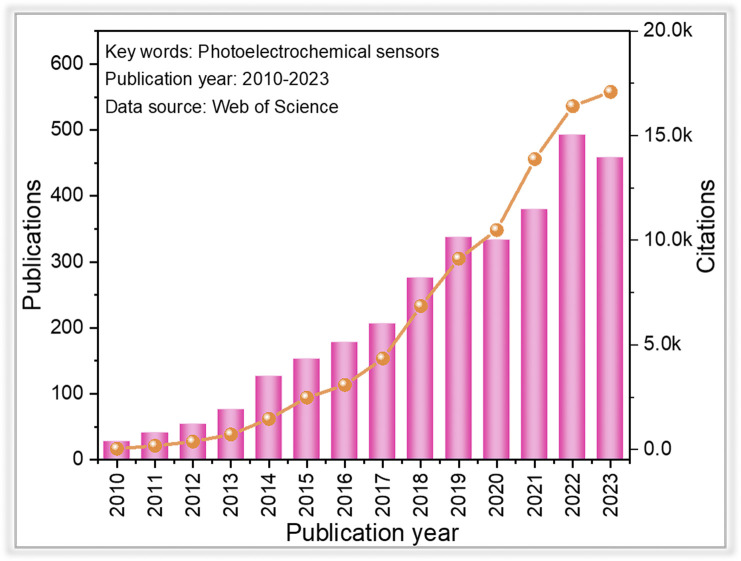
Overview of the research contributions in photoelectrochemical sensors since 2010.

Recently, photoelectrochemical (PEC) detection methods, as a branch of electrochemical (EC) detection techniques, have gained much attention in the field of tracing environmental pollutants,^12–15^ which can be seen from the number of publications since 2010 (Fig. 1). Compared with conventional electrochemical detection techniques, the PEC method is based on the photo-induced electron–hole pair redox properties, which make it possible to generate signals (photocurrent).^16–18^ Since light and photocurrent are employed as the excitation source and identification signal, respectively, a PEC sensor handles relatively lower background noise and higher sensitivity than those of conventional electrochemical detection methods due to the difference in the energy form of the excitation source and the converted electrical signal. Moreover, the PEC sensor is not directly in contact with the measured object, resulting in less influence from the measurement condition.^19^ These advantages contribute to the simplicity of instrumentation, low cost, and ease of miniaturization associated with the PEC detection method.^20–23^

**Fig. 2:**
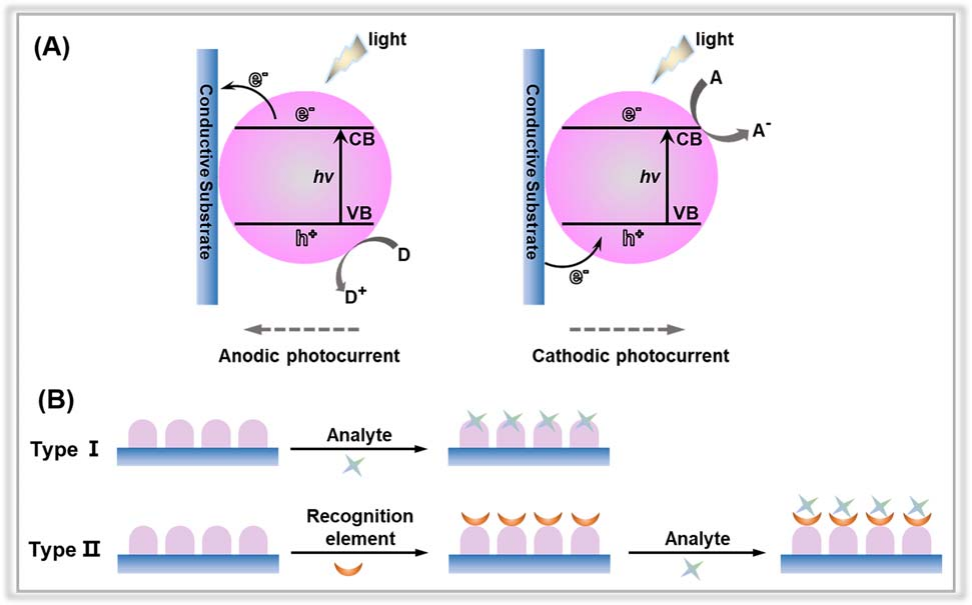
(A) Anodic and cathodic photocurrent generation mechanisms of photoactive material-based electrodes. (B) Different types of PEC sensors with or without the recognition elements.

In a typical PEC sensing system, the incoming light is regarded as the excitation source, and the electrical signal is used as the detection signal.^18,24,25^ When exposed to light, the electrode containing the photoactive material is excited, resulting in the generation of photogenerated electron–hole pairs, which are subsequently transferred to the electrode surface. Cathodic photocurrent results from the electrons on the condition band (CB) being trapped by electron acceptors (A); otherwise, the holes on the valence band (VB) transfer to the surface of photoactive materials, where they react with the electron donors (D) to form anodic photocurrent (Fig. 2A).^17^ According to the different generation processes of the photocurrents at the interface between the electrode and electrolyte, the PEC detection sensors can be classified into two categories: (1) the analytes serve as electron donors or acceptors to directly react with photoactive materials;^26,27^ (2) the electrodes are first modified by recognition elements, after which the target concentration is determined through indirect physicochemical interactions between the targets and the recognition elements (Fig. 2B). For the commonly used recognition elements used in the PEC detection system, they can be divided into three categories: (1) aptamers refer to single-stranded synthetic nucleic acid molecules (DNA or RNA);^28–30^ (2) antibodies, which are mainly proteins, can be used as recognition elements to bind with target antigens such as proteins, toxins, and pathogens;^31^ (3) enzymes are biological catalysts that can bind to and act upon specific substrates.^32^

As can be seen from the inset above, whether the recognition element is contained or is free on photoelectrochemical sensing platforms, photoelectrochemical detection performance of a fabricated electrode is mainly determined by three factors, including light absorption capacity, photogenerated charge carrier separation and transportation within photoactive materials, and the transfer of surface charge carrier to the detection species.^33^ To achieve excellent detection activity with PEC sensors, researchers have focused on exploiting various sensing devices and detection modes, and the design engineering of photoactive materials. Much effort has been directed towards reducing costs and simplifying the device fabrication for versatile and portable PEC sensing platforms. For instance, in some detection devices, capacitors and digital multimeters are utilized to output electrical signals instead of electrochemical workstations.^34–36^ Another area of research involves exploring different detection patterns, such as split-type detection, self-power detection, visual detection, and high-throughput detection. Despite extensive efforts in designing detection devices and modifying detection modes, the primary focus in constructing PEC platforms has been on investigating photoelectrodes.^19,37^ To date, significant effort has been devoted to the development of photoactive materials as electrodes for PEC sensors. These materials include metal oxides, metal organic frameworks, graphene and carbon nitride.^38,39^

**Fig. 3:**
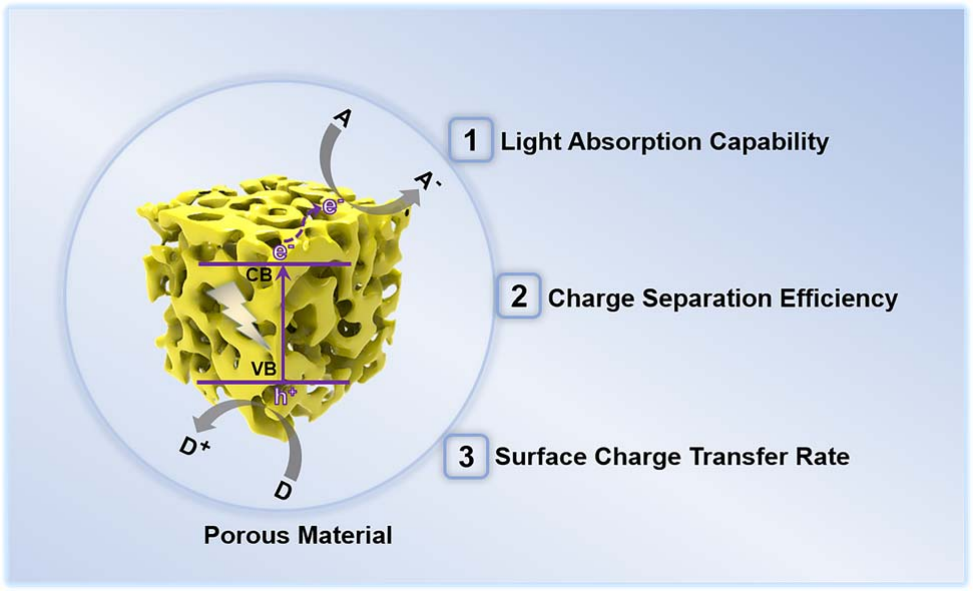
Structural effects of photoactive porous materials on the performance of photoelectrochemical detection of environmental pollutants.

In recent years, porous materials have received considerable attention owing to their high surface area, relatively low density, large accessible space, and variable chemical composition.^40–42^ Research interests in the applications of porous material-based PEC sensors were rapidly increasing, particularly in the field of determining environmental pollutants.^43^ Due to their unique structure, porous materials are preferred over bulk materials for photoelectrochemical determination of environmental contaminants. As illustrated in Fig. 3, the porous material has a structural influence on the three factors governing the PEC detection activity of the sensor.

(1) Light absorption capability: porous materials with small pores enable the incident light to penetrate their pore walls and scatter intensely inside the pore channels, thereby greatly improving the light harvesting ability of the porous materials.^44^ Further, the feasible modifications of the band structure of the porous materials by altering the pore diameter also promote light absorption, particularly in the visible light region.

(2) Photogenerated charge carrier separation and transport efficiency: the photoelectric conversion efficiency of a photoelectrode depends on the separation and transport of photogenerated charge pairs within the photoactive material. In the case of bulk materials, photogenerated charge pair separation normally occurs in the space charge layer and the adjacent layers, leading to easy recombination. However, for porous materials, the charge separation and migration paths can be shortened due to their unique porous structures,^45^ enabling efficient photogenerated charge transfer within porous materials.

(3) Surface charge carrier transfer rate: porous materials possess a large specific surface area, which is advantageous for mass transfer during the photoelectrochemical detection. In addition, the exposed surface areas with abundant active sites provide sufficient functional groups conducive to interaction with recognition elements or direct trapping of the probe species. Hence, porous materials exhibit rapid surface charge carrier transfer rates and reaction kinetics.

Thus, photoelectrochemical sensing platforms based on porous materials have remarkable detection capabilities against environmental contaminants. This can be attributed to several factors, including enhanced light absorption, high photogenerated charge carrier separation and transport efficiency, and rapid surface charge carrier transfer rates during photoelectrochemical detection. As a result, these platforms offer a wide linear range, low detection limits, high sensitivity, excellent selectivity, and outstanding stability.

Recently, a significant number of reviews on PEC detection sensors for environmental pollutant determination have been published.^11,16,46–50^ However, these studies focused on strategies for fabricating PEC detection systems and identifying the multiple contaminants, neglecting to discuss the effect of the presence of porous materials on the detection activity of PEC sensors. For instance, in 2020, Shu *et al.* provided a comprehensive summary of current research in photoelectrochemical sensing, with a particular emphasis on material design and engineering to regulate photoelectrochemical sensing performance. They also discussed photoelectrochemical sensing devices and detection modes.^33^ In another review by Li and co-workers, the central theme was a comparative analysis of electrochemical detection and photoelectrochemical detection, specifically addressing the question of which analytical method is more effective for tracing environmental pollutants.^11^ Further, in 2021, Yan *et al.* provided a concise overview of the fundamental and research progress of functional materials (such as metals, metal oxides, inorganic 2D materials, and carbon nanomaterials) in electrochemical and photoelectrochemical technologies for monitoring environmental pollutants.^51^ Despite these efforts, few reviews have focused on the relationship between the structure of porous materials and the detection activity of associated PEC sensors. So, in this review, we seek to thoroughly investigate the recent advancements in porous materials for environmental contaminant detection. Indeed, porous materials encompass a wide range of different types. Here, we specifically focus on photoactive porous materials, including but not limited to metal oxides, metal–organic frameworks (MOFs), covalent organic frameworks (COFs), graphitic carbon nitride (g-C_3_N_4_), and MXene (Table 1). We extensively discuss the structural effects of porous materials on the performance of PEC sensors. Finally, we propose the main challenges and future prospects of porous materials in the realm of PEC detection sensors. However, due to the space limitations of this review and the widespread use of porous materials in the field of PEC detection sensors, many intriguing and significant studies may not have been addressed. We apologize for any inadvertent omissions and appreciate the valuable contributions of all researchers in this field.

**Table 1:** Typical photoactive porous materials for the photoelectrochemical monitoring of environmental contaminants

Photoactive porous materials	Common preparation method	Advantages
Metal oxides	Anodic oxidation, chemical bath deposition, hydrothermal	Suitable *E*_g_, fast surface kinetics
Metal–organic frameworks	Hydrothermal, slow evaporation and diffusion, iono-thermal, microwave-assisted, mechanochemical, electrochemical, sonochemical, microemulsion	Unique porosity, adjustable light response range
Covalent organic frameworks	Hydrothermal, ionothermal, microwave-assisted, sonochemical, mechanochemical, light-induced processes	π-electron conjugation, low *E*_g_
Graphitic carbon nitride	Chemical exfoliation, thermal oxidation	Metal-free nontoxic, visible light responsive
MXene	Etching method	Small diffusion barrier, high conductivity

## 2. Photoactive porous materials

### 2.1. Metal oxides

**Fig. 4:**
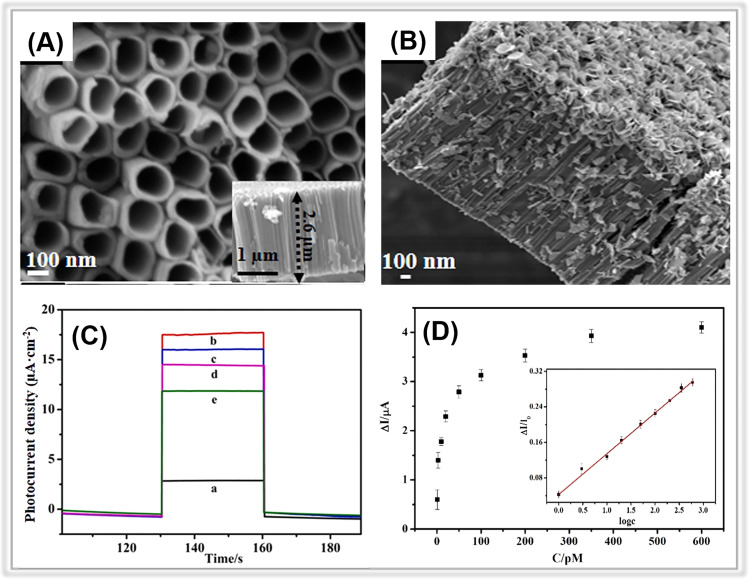
SEM images of (A) TiO_2_ NTs and (B) BiOI NFs/TiO_2_ NTs. (C) Photocurrent responses of (a) TiO_2_ NTs, (b) BiOI NFs/TiO_2_ NTs, (c) aptamer/BiOI NFs/TiO_2_ NTs, (d) BSA/aptamer/BiOI NFs/TiO_2_ NTs, and (e) atrazine/BSA/aptamer/BiOI NFs/TiO_2_ NTs. (D) Photocurrent change against atrazine concentrations. Adopted from ref. 67. Copyright 2021, with permission of Elsevier.

Metal oxides play a crucial role in the field of photoelectrochemical sensing.^52,53^ Indeed, there is a large body of research focusing on porous metal oxides for PEC sensing, which is difficult to categorize in a simple way, so here we only present some representative porous metal oxides, in particular semiconductor metal oxides (Table 2). Among all the porous metal oxides applied in PEC sensing, TiO_2_ has always occupied a unique position.^54–59^ In addition, PEC detection sensors have been constructed using ZnO, SnO_2_, WO_3_, and Fe_2_O_3_ (ref. 60–65) (Table 3). For instance, in a study by Tavella and co-workers, a porous TiO_2_ array modified titanium electrode was constructed for PEC sensing of dopamine.^66^ The as-prepared electrode performed well for dopamine and has a wide response range (200∼1500 μM) and a low detection limit (20 μM). In addition to nanoarrays, nanotubes also have a large number of pores. For instance, Fan *et al.* prepared a BiOI nanoflowers/TiO_2_ nanotubes (BiOI/TiO_2_) composite through a hydrothermal method (Fig. 4A and B).^67^ Due to the presence of porous nanotube structure, the BiOI NFs/TiO_2_ NTs electrode provided more spaces for anchoring the aptamer, and thus the PEC platform demonstrated a significant activity for atrazine determination with a low detection limit of 0.5 pM (Fig. 4C and D).

**Table 2:** Features of different metal oxides used in the fabrication of photoelectrochemical detection platforms

Photoactive metal oxides	Band gap/eV	Preparation method
TiO_2_	3.0∼3.3	Anodic oxidation, hydrothermal, sol–gel
ZnO	3.7	Electrodeposition, hydrothermal, chemical bath deposition
SnO_2_	3.6∼4.0	Hydrothermal, electrospun
WO_3_	2.8	Hydrothermal, solvothermal, co-precipitation
Fe_2_O_3_	2.2	Electrodeposition, hydrothermal

**Table 3:** Metal oxide-based photoelectrochemical detection sensors for tracing environmental contaminants^a^

Working electrode	Analyte	LOD	Linear concentration range	Ref.
NiCo-LDHs/TiO_2_ TNAs	Cr(vi)	0.12 μM	0.5 μM∼1.8 mM	55
CdSe/TiO_2_ TNAs	Cd^2+^	0.35 nM	1 nM∼10 mM	72
Fe^3+^/ZnO-Ag	Hg^2+^	0.1 nM	0.5 nM∼100 nM	60
WO_3_/Au	Hg^2+^	2.0 pM	4.2 pM∼840 pM	61
Fe_2_O_3_-CdS	Cu^2+^	0.5 nM	50 nM∼600 μM	73
ZnO-CdS	Cu^2+^	3 nM	0.01 μM∼1 mM	69
CdS/ZnO	Cd^2+^	3.3 μM	0.01 mM∼5 mM	74
BiOI/ZnO NRs	Pb^2+^	7.5 μM	10 μM∼100 μM	75
MIP@TiO_2_ NTAs	Perfluorooctane sulfonate	86 ng mL^−1^	0.5 μM∼10 μM	76
CdS/MnO_2_	Paraoxon	0.017 ng mL^−1^	0.05 ng mL^−1^∼10 ng mL^−1^	77
TiO_2_ TNAs/CdS	Bisphenol A	0.5 pM	1 pM∼100 pM	78
SnO_2_	Bisphenol A	1.2 nM	2 nM∼500 nM	79
MoS_2_/ZnO	Propyl gallate	12 nM	0.125 μM∼1.47 μM	80
TiO_2_ array/Ti	Dopamine	20 μM	0.2 mM∼1.5 mM	66
CdS QDs/TiO_2_ TNAs	Asulam	4.1 pg mL^−1^	0.02 ng mL^−1^∼2.0 ng mL^−1^	57
BiOI/TiO_2_ NTAs	Atrazine	0.5 pM	1.0 pM∼600.0 pM	67
Bi_2_S_3_/V_o_-TNTAs	Chlorpyrifos	6 nM	0.07 μM∼3.0 μM	68
GQDs/TiO_2_ TNAs	Chloramphenicol	57.9 pM	0.5 nM∼100 nM	81
WO_3_/CuMnO_2_	Nitrofurazone	1.19 nM	0.015 μM∼32 μM	70
Bi_2_WO_6_/α-Fe_2_O_3_	Tetracycline	0.3 μM	0.01 μM∼25 μM	63
P(33DT-*co*-3TPCA)/[BMIM]Cl-ZnO NRs	Aflatoxin B1	0.058 ng mL^−1^	0.10 ng mL^−1^∼10 ng mL^−1^	82
rGO/TiO_2_ TNAs	Microcystin-LR	0.5 fM	1.0 fM∼500 fM	83
Cu_2_O/TiNTs	Sulfide	0.6 μM	1 μM∼300 μM	84
SnO_2_-AuNPs	Nitrite	0.48 nM	1 nM∼100 μM	85

a MIP: molecularly imprinted polymer; NTAs: nanotubes; NRs: nanorods; V_o_-TNTAs: TiO_2_ nanotube arrays with oxygen vacancies; TiNTs: TiO_2_ nanotube arrays; GQDs: graphene quantum dots; P(33DT-*co*-3TPCA): poly(3,3′-dithiophene-*co*-3-thiophenecarboxylic acid); [BMIM]Cl: 1-butyl-3-methylimidazolium chloride.

Similarly, Wu *et al.* developed a PEC sensor for chlorpyrifos (CPF) detection based on bismuth sulfide nanoparticles decorating TiO_2_ nanotubes with oxygen vacancies (Bi_2_S_3_/V_o_-TNTAs), which realized a rapid photocurrent response to CPF within a linear range of 0.07∼3.0 μM.^68^

In addition to the porous TiO_2_ nanomaterials, other photoactive porous metal oxides (ZnO, SnO_2_, WO_3_, and Fe_2_O_3_) have also exhibited excellent photoelectrochemical detection activity towards environmental pollutants. In a study by Wu and co-workers, CdS nanocrystals were decorated on porous one-dimensional (1D) ZnO nanorods through a pulsed electrodeposition technique.^69^ The resulting CdS/ZnO hybrid photoelectrode was employed for PEC detection of the heavy metal ion, Cu^2+^. The porous ZnO nanorods provided an enlarged surface area for the dispersion of nano-sized CdS nanocrystals, thereby enhancing the photogenerated electron transfer *via* the “1D electron highway”. The CdS/ZnO composite electrode demonstrated an ultrasensitive LOD of approximately 3 nM within a wide linear range of 0.01∼1000 μM. Meanwhile, Velmurugan *et al.* prepared a WO_3_/CuMnO_2_ p–n heterojunction composite and applied it for “signal-on” PEC sensing of nitrofurazone.^70^ Porous WO_3_ nano-tiles were synthesized *via* a hydrothermal method and coupled with CuMnO_2_ nanoparticles by an evaporative impregnation method. The porous WO_3_ provided abundant surface sites for interaction with CuMnO_2_ nanoparticles. Further, a p–n heterojunction was formed between the p-type CuMnO_2_ and the n-type WO_3_, which promoted photogenerated electron transfer due to the presence of a built-in electric field.^71^ As a result, the PEC nitrofurazone sensing performance of the WO_3_/CuMnO_2_ composite electrode demonstrated a wider detection range of 0.015∼32 μM with a lower LOD of 1.19 nM, compared to pure CuMnO_2_ nanoparticles.

α-Fe_2_O_3_ is a well-known visible light active semiconductor with a band gap of 2.2 eV, making it suitable for applications in PEC sensing. In 2019, Adhikari *et al.* prepared a Bi_2_WO_6_/α-Fe_2_O_3_ heterojunction photoelectrode by depositing porous α-Fe_2_O_3_ layers on the Bi_2_WO_6_ nanoflakes.^63^ The influence of the α-Fe_2_O_3_ layer thickness on the PEC detection activity of tetracycline was thoroughly investigated. After introducing porous α-Fe_2_O_3_ layers, the photocurrent of the hybrid composite-based photoelectrode increased (4.3 μA cm^−2^) compared to that of pristine Bi_2_WO_6_ (1.2 μA cm^−2^). Under optimized conditions, the photoelectrode demonstrated a LOD of around 0.3 μM in the range from 0.01 μM to 25 μM. Hence, PEC sensors based on porous metal oxides hold great potential for tracing environmental contaminants.

### 2.2. Metal–organic frameworks

Metal–organic frameworks (MOFs), which are potential porous materials composed of transition metal ions and organic ligands, have attracted much attention in the area of environmental pollutants determination.^86,87^ MOFs, particularly photosensitive MOFs, have many potential applications in PEC sensing due to their inherent characteristics, such as permanent porosity, chemical stability, and unique optical properties.^39,88–91^ The Zeolitic Imidazolate Framework (ZIF), Materials Institute Lavoisier frameworks (MILs), Universitetet i Oslo (UiO), copper(ii) benzene-1,3,5-tricarboxylate (Cu-BTC), and porphyrin-based MOFs (PCN) series MOFs have been extensively investigated for PEC sensing applications against hazardous pollutants (Table 4).^92^

**Table 4:** Metal–organic framework-based photoelectrochemical detection sensors for tracing environmental pollutants^a^

Working electrode	Analyte	LOD	Linear concentration range	Ref.
Eu-MOF	Fe^3+^	0.0899 μM	1 μM∼100 mM	104
Cu-MOF-NH_2_	Kanamycin	0.1 nM	0.5 nM∼650 nM	105
NiPc-Ni MOF	Curcumin	0.8 nM	2.5 nM∼16 μM	106
Cu-BTC/g-C_3_N_4_	Glyphosate	0.13 pM	1 pM∼10 nM	100
Cu-BTC@Cu_2_O	Dioctyl phthalate	9.15 pM	25.0 pM∼0.1 μM	107
PCN-224/rGO	*p*-arsanilic acid	5.47 ng L^−1^	10 ng L^−1^∼10 mg L^−1^	108
CdS QDs/PCN-224	Doxorubicin hydrochloride	3.57 nM	10 nM∼1 μM	102
	Gentamicin sulfate	0.158 nM	1 nM∼1 μM	
PCN-222@g-C_3_N_4_	Kanamycin sulfate	0.127 nM	1 nM∼100 nM	101
Ce-Por-MOFs/AgNWs	Ronidazole	0.038 nM	0.1 nM∼104 nM	109
ZIF-8@ZnIn_2_S_4_	Tetracycline	0.1 pM	1 pM∼700 pM	95
MIP@NH_2_-MIL-125(Ti)/TiO_2_	Oxytetracycline	60 pM	0.1 nM∼10 μM	97
CdS/Eu-MOF	Ampicillin	0.093 nM	0.1 nM∼0.2 μM	110
[Ru(bpy)_3_]^2+^@Ce-UiO-66/Mn:Bi_2_S_3_	Ofloxacin	6 pM	0.01 nM∼100 nM	98
g-C_3_N_4_/Au/NH_2_-UiO-66	d-penicillamine	0.0046 μM	10 nM∼400 μM	99
Er-MOF@AuNPs	Aflatoxin B1	19.6 fg mL^−1^	0.005 ng mL^−1^ ∼ 10.0 ng mL^−1^	111

a Eu-MOF: europium(iii)-based metal organic framework; Cu-MOF-NH_2_: copper(ii) 2-aminoterephthalic acid; NiPc-Ni MOF: nickel phthalocyanine-based metal organic framework; Cu-BTC: copper(ii) benzene-1,3,5-tricarboxylate; Ce-Por-MOFs: Ce-porphyrin-metal–organic frameworks; NH_2_-MIL-125(Ti): amino-functionalized titanium(iv) based MOFs; ZIF-8: zeolitic imidazolate framework-8; CdS QDs: CdS quantum dots; PCN-222, PCN-224: zirconium-porphyrin metal–organic framework; Ce-UiO-66: Ce doped zirconium-based MOF with terephthalic acid ligands; NH_2_-UiO-66: zirconium-based MOF with 2-aminoterephthalic acid ligands; Er-MOF: erbium(iii)-based metal organic framework.

#### 2.2.1. ZIF

**Fig. 5:**
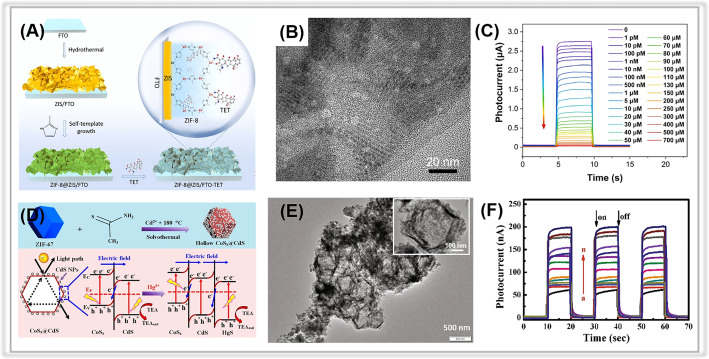
(A) The preparation of a ZIF-8@ZIS-based PEC sensor for tetracycline. (B) TEM image of ZIF-8@ZIS. (C) The corresponding calibration curve for the photocurrent signal responses toward various concentrations of tetracycline. Adopted from ref. 95. Copyright 2022, with permission of Elsevier. (D) The synthetic process of hollow CoS_*x*_@CdS composites and the band structures of CoS_*x*_@CdS/HgS composites and charge separation under visible light irradiation. (E) TEM image of CoS_*x*_@CdS composites. (F) Photocurrent responses of the CoS_*x*_@CdS-modified electrodes in the presence of Hg^2+^ of different concentrations. Adopted from ref. 103. Copyright 2020, with permission of Elsevier.

The ZIF family of MOFs has been widely employed in the fabrication of PEC detection sensors. Among them, zeolitic imidazolate framework-8 (ZIF-8), a typical member of MOF materials, is composed of zinc ions and 2-methyl-imidazole ligands. ZIF-8 possesses excellent stability in aqueous conditions.^93,94^ More importantly, ZIF-8 exhibits porous structures and has the ability to generate abundant reactive species under light irradiation. These unique properties make it suitable for constructing PEC detection platforms for environmental pollutant determination. For instance, Chen *et al.* prepared a ZIF-8/ZnIn_2_S_4_ (ZIF-8@ZIS) photoelectrode for PEC detecting tetracycline (Fig. 5A).^95^ In this detection system, the porous ZIF-8, with plenty of active Zn(ii) sites, could interact rapidly and specifically with the tetracycline, resulting in quenched photocurrent due to the swift transfer of photoelectrons. The lowest detection limit calculated for the ZIF-8@ZIS PEC sensor was *ca.* 0.1 pM (Fig. 5B and C). In addition, it exhibited exceptional speediness, high stability, and strong selectivity during PEC monitoring of tetracycline.

#### 2.2.2. MIL

Similar to the ZIF series MOFs, the MIL series MOFs have also been utilized in PEC detection. Recently, NH_2_-MIL-125(Ti), a titanium-based metal organic framework (Ti-MOF) derived from the Ti metal ions and NH_2_-BDC ligands, has exhibited excellent visible light absorption ability and stable redox reaction properties.^96^ In 2021, Yang *et al.* developed a molecularly imprinted NH_2_-MIL-125(Ti)/TiO_2_ composite-based photoelectrochemical sensor for oxytetracycline detection.^97^ The NH_2_-MIL-125(Ti)/TiO_2_ hybrid composite was synthesized *via* a simple solvothermal method, and then a molecularly imprinted polymer (MIP) was modified as a recognition element. The photocurrent of the MIP@NH_2_-MIL-125(Ti)/TiO_2_-modified electrode was significantly enhanced (0.8 μA) compared to that of the MIP@TiO_2_ (0.1 μA), owing to the improved visible light absorption capacity and well-matched band levels of the two components. As a result, the MIP@NH_2_-MIL-125(Ti)/TiO_2_-based photoelectrochemical detection sensor demonstrated a wide linear detection range from 0.1 nM to 10 μM, with a LOD of 60 pM.

#### 2.2.3. UiO

UiO series MOFs have also demonstrated excellent photoelectrochemical detection activities against environmental contaminants. For instance, UiO-66 is composed of the Zr_6_O_4_(OH)_4_ cluster and terephthalic acid. In 2021, Feng *et al.* synthesized [Ru(bpy)_3_]^2+^@Ce-UiO-66/Mn:Bi_2_S_3_ composites and utilized them to construct aptamer-based PEC sensors for ofloxacin (OFL) detection.^98^ Ce-UiO-66 MOFs were obtained by doping UiO-66 with Ce-elements. The cycling of Zr^4+^–Zr^3+^ and Ce^4+^–Ce^3+^ in porous Ce-UiO-66 enhanced charge separation efficiency. Additionally, the [Ru(bpy)_3_]^2+^ broadened the range of light absorption, and Mn:Bi_2_S_3_ acted as a photosensitizer, improving the separation efficiency of photogenerated charge pair. The photocurrent response of [Ru(bpy)_3_]^2+^@Ce-UiO-66/Mn:Bi_2_S_3_ composite was improved in comparison to Mn:Bi_2_S_3_. Thus, the composite-modified photoelectrodes showed excellent photoelectrochemical detection activity (concentration range: 0.1∼100 nM, LOD: 6 pM). Similarly, in a study by Wu and co-workers, a photoelectrochemical sensor based on Au NPs loaded on porous g-C_3_N_4_ nanosheets and hexagonal NH_2_-UiO-66 composite (g-C_3_N_4_/Au/NH_2_-UiO-66) was constructed for the detection of d-penicillamine.^99^ The presence of the Z-scheme heterojunction in g-C_3_N_4_/Au/NH_2_-UiO-66 promoted the photogenerated electron transfer, and the strong binding between Au NPs and d-penicillamine enhanced the selectivity and sensitivity of the sensor. The composite-modified electrode exhibited a maximum photocurrent about 10 times larger than that of g-C_3_N_4_. Thus, the proposed g-C_3_N_4_/Au/NH_2_-UiO-66-based photoelectrode demonstrated a low detection limit of 0.0046 μM in a wide linear range from 10 nM to 400 μM.

#### 2.2.4. Cu-BTC

In addition to the aforementioned MIL, ZIF, and UiO series MOFs, several other MOFs, such as Cu-BTC (HKUST-1), have been employed in the fabrication of PEC sensing platforms. As for Cu-BTC, it consists of copper ions and benzene-1,3,5-tricarboxylate ligands. In 2019, Cao *et al.* constructed a PEC sensor for glyphosate assays utilizing a porous Cu-BTC/g-C_3_N_4_ composite.^100^ Porous Cu-BTC offered the benefit of trapping glyphosate molecules and significantly enhanced the photoelectric conversion efficiency of g-C_3_N_4_ nanosheets. In this detection system, the formation of the Cu-glyphosate complex increased the steric hindrance to electron transfer between the composite and the electrode surface, leading to a reduction of photocurrent. Thus, a correlation between photocurrent and glyphosate concentration was established over a wide range from 1 pM to 1 nM.

#### 2.2.5. PCN

PCN is a type of MOF comprising zirconium (Zr) clusters and porphyrin ligands. Its remarkable stability and photoelectrochemical properties make it highly promising for the fabrication of PEC platforms. For instance, Dong and co-workers constructed a photoelectrochemical sensor for the identification of kanamycin sulfate based on a PCN-222@g-C_3_N_4_ composite.^101^ Through a physical microwave mixing process, g-C_3_N_4_ with a broad bandgap was effectively coupled with porous, narrow-band gap PCN-222. The composite-modified photoelectrode demonstrated a low detection limit of 0.127 nM with a wide linear range from 1 nM to 1000 nM. Similar work has also been conducted employing the CdS QDs/PCN-224 composite.^102^ Sub-3 nm CdS quantum dots were uniformly distributed in the porous PCN-224. Compared to pure PCN-224 and CdS QDs, the photocurrent response of the composite-based electrode was improved. With regard to the detection of doxorubicin hydrochloride and gentamicin sulfate, the LODs were as low as 3.57 nM and 0.158 nM, respectively.

#### 2.2.6. Ln-MOFs

Some other Ln-MOFs have also been employed in the fabrication of PEC sensing platforms. Eu-MOFs, as a representative class of Ln-MOFs, possess excellent light-harvesting properties. For instance, Gao *et al.* prepared a CdS nanoparticle/europium metal–organic framework (CdS/Eu-MOF) composite and utilized it for PEC monitoring of ampicillin.^110^ The porous structure of the Eu-MOF enhanced the light absorption capability of the composite while effectively inhibiting the recombination of photogenerated charge pairs. The CdS/Eu-MOF-based PEC sensor achieved a low detection limit of around 0.093 nM, along with great selectivity, outstanding repeatability, and desired stability. In summary, porous metal–organic frameworks have demonstrated remarkable potential for photoelectrochemical sensing of environmental toxic species.

#### 2.2.7. MOF derivatives

Moreover, MOF derivatives have shown potential for identifying toxic species in the environment^116,120,123^ (Table 5). For instance, Zhang *et al.* prepared a hollow CoS_*x*_@CdS polyhedron composite for photoelectrochemical Hg^2+^ assays. The composites were obtained by decorating CdS nanoparticles on the surface of CoS_*x*_ through a simple solvothermal method, using a zeolitic imidazolate framework-67 (ZIF-67) as the sacrificial template and cobalt precursor^103^ (Fig. 5D). The porous CoS_*x*_ polyhedron component in the composite facilitates the transfer of photogenerated electrons during the PEC detection process (Fig. 5E). The photocurrent of the CdS/CoS_*x*_ composite-modified electrode was about 50 nA, 2.5 times higher than that of CdS. Photoluminescence spectra (PL) and electrochemical impedance spectroscopy (EIS) measurements also confirmed its enhanced photogenerated charge pair separation ability. Compared to the CdS or CoS_*x*_ modified electrodes, the CdS/CoS_*x*_ modified electrode exhibited an increasing photocurrent in the presence of analyte-Hg^2+^ due to the *in situ* formation of a Z-scheme CoS_*x*_@CdS/HgS *via* a selective ion-exchange reaction. A strong linear relationship was established between the photocurrent and log(Hg^2+^) concentrations in the range of 0.005 nM to 1000 nM, with a LOD of 0.002 nM (Fig. 5F).

**Table 5:** Derivatives of metal–organic framework based photoelectrochemical detection sensors for environmental pollutant assay^a^

Working electrode	Template MOF	Analyte	LOD	Linear concentration range	Ref.
CoS_*x*_@CdS	ZIF-67	Hg^2+^	0.002 nM	0.01 nM∼1 μM	103
Cu_2_O/CuO/TiO_2_	NH_2_-MIL-125(Ti)	Pb^2+^	6.8 fM	10 fM∼1 μM	112
CdCoS_2_	ZIF-67-S	Chlorpyrifos	0.57 ng mL^−1^	0.001 μg mL^−1^∼270 μg mL^−1^	113
MIPs@TiO_2_-C	MIL-125(Ti)	Ofloxacin	2.91 pg mL^−1^	0.01 ng mL^−1^∼3 μg mL^−1^	114
CuO	Cu-BTC	Malathion	0.086 nM	0.1 nM∼104 nM	115
ZnO-Co_3_O_4_	Zn-BTC	Sulfadiazine	1.2 nM	0.005 μM∼18.5 μM	116
ZnO/g-C_3_N_4_	ZIF-8	Oxytetracycline	1.49 fM	5 fM∼200 nM	117
ZnIn_2_S_4_@TiO_2_	ZIF-8	Lincomycin	0.084 pM	0.1 pM∼0.1 nM	118
ZnCdS@MoS_2_	ZIF-8	Lincomycin	0.076 nM	0.1 nM∼300 nM	119
AuNPs/In_2_O_3_@g-C_3_N_4_	MIL-68(In)	Tetracycline	3.3 pM	0.01 nM∼0.5 μM	120
Zn_*x*_Co_3−*x*_O_4_/N-GQDs/AgBiS_2_	ZnCo-ZIF	Ampicillin	0.25 pM	0.5 pM∼10 nM	121
In_2_O_3_–In_2_S_3_–Ti_3_C_2_ MXene	MIL-68(In)	Microcystin-LR	0.169 pM	0.5 pM∼400 nM	122

a ZIF-67: zeolitic imidazolate framework-67; MIPs: molecularly imprinted polymers; ZIF-8: zeolitic imidazolate framework-8; MIL-125(Ti): titanium(iv) terephthalic acid; Cu-BTC: Cu-BTC: copper(ii) benzene-1,3,5-tricarboxylate; Zn-BTC: Zn(ii) benzene-1,3,5-tricarboxylate; MIL-68(In): In(iii) 1,4-benzenedicarboxylate; ZnCo-ZIF: bimetallic ZnCo-zeolitic imidazolate framework.

Derivatives of the MIL family of MOFs have also demonstrated excellent PEC detection activities against environmental toxic species. In a study by Zhang and co-workers, COOH-functionalized TiO_2_ (TiO_2_-C) was achieved *via* one-step calcination of MIL-125(Ti).^114^ Due to the large specific surface area and abundant functional groups of TiO_2_-C, it demonstrated superior photochemical, electrochemical, and PEC detection performance compared to MIL-125(Ti). Furthermore, it was also useful for grafting molecularly imprinted polymers (MIPs). The MIPs@TiO_2_-C, with a large number of binding sites, provides precise electron transfer channels, resulting in improved sensitivity and selectivity for antibiotics such as ofloxacin. Under optimal conditions, the prepared sensor has a low detection limit (2.9 pg mL^−1^) and a wide linear concentration range (0.01 ng mL^−1^∼3 μg mL^−1^).

MIL-68(In) is also an ideal template for obtaining porous photoactive materials. For instance, Yan *et al.* synthesized an In_2_O_3_–In_2_S_3_–Ti_3_C_2_ MXene composite on the base of MIL-68(In)-derived In_2_O_3_ hollow tubular^122^ and then used it to construct a dual-mode (photoelectrochemical and photofuel cell) self-powered apta-sensing platform for detecting microcystin-LR (MC-LR). Porous In_2_O_3_ hollow tubulars with a large specific surface area provide abundant active sites, while the well-matched energy levels of In_2_O_3_ and In_2_S_3_ and the Ti_3_C_2_ MXene quantum dots acting as electron transfer mediators both accelerate the separation of photogenerated charge carriers. This sensing platform revealed excellent PEC detection activity in the range from 0.5 pM to 400 nM, with a LOD of 0.169 pM. In another research, Feng *et al.* used MIL-68(In) as the precursor for fabricating homogeneous In_2_O_3_ nanoparticles through high temperature calcination in an air atmosphere.^120^ The formation of a heterojunction between the porous In_2_O_3_ and g-C_3_N_4_ facilitated the separation and transfer of photogenerated charge pairs. The introduction of gold nanoparticles (Au NPs) with the localized surface plasmon resonance (LSPR) effect also improved visible light absorption and photoelectron transfer. The photocurrent of the Au NPs/In_2_O_3_@g-C_3_N_4_ composite reached 1.75 μA, much larger than that of pure g-C_3_N_4_. The Au NPs/In_2_O_3_@g-C_3_N_4_ was successfully applied to fabricate a label-free photoelectrochemical apta-sensing platform for tetracycline detection, which yielded a wide linear range from 0.01 nM to 500 nM with a LOD of 3.3 pM. Porous composites derived from Cu-BTC or Zn-BTC have also demonstrated great potential for PEC detection. In a study by Cao *et al.*, Cu-BTC MOF (BTC: benzene-1,3,5-tricarboxylic acid) was calcinated to achieve porous hierarchical CuO.^115^ The CuO material has a large specific surface area, which is favorable for the capture of the target species, malathion. The facile PEC detection sensor achieved a LOD of 0.086 nM in the range of 0.1 nM to 104 nM.

### 2.3. Covalent organic frameworks

**Fig. 6:**
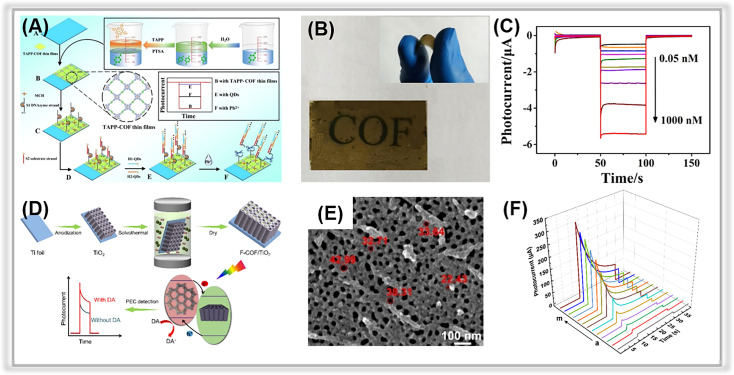
(A) Illustration of the TAPP-COF-based PEC sensor for tracing Pb^2+^. (B) The flexible photograph of TAPP-COF thin films. (C) Photocurrent response of the photoelectrochemical detection sensor to different concentrations of Pb^2+^. Adopted from ref. 124. Copyright 2021, with permission of American Chemical Society. (D) The construction of the F-COF/TiO_2_ NTA platform for PEC sensing for dopamine. (E) SEM image of F-COF/TiO_2_ NTA. (F) Photocurrent response of F-COF/TiO_2_ NTA to different concentrations of dopamine. Adopted from ref. 125. Copyright 2021, with permission of American Chemical Society.

Like metal–organic frameworks, covalent organic frameworks (COFs) are a new class of porous materials that have attracted much interest for constructing the PEC analytic platforms.^128,129^ Generally, COFs are composed of covalently bonded light elements, such as C, N, H, O, B, and Si. The porous nature of COFs favors the trapping of probe species such as heavy metal ions. Moreover, the electronic interactions between the COF layers are realized by the π-stacked aromatic subunits, providing sufficient channels for charge transport.^130–132^ So far, several COFs have been employed in the field of PEC detection, such as D-TA COF,^133,134^ TAPP-COF,^124^ TTPA-COF,^135^ F-COF,^125^ p-bqy-COF,^136^ and PFA-130.^137^ However, the studies on PEC sensors based on covalent organic frameworks for tracing environmental pollutants are still in their early stages, as summarized in Table 6. In a study by Zhao and co-workers, a porphyrin-based covalent organic framework thin film (TAPP-COF) was synthesized *via* a liquid–liquid interfacial method and used as a photocathode material for photoelectrochemical “on-off-on” sensing of Pb^2+^.^124^ Due to the unique charge channels of porous COFs and the excellent photoelectric properties of porphyrin,^138^ the TAPP-COF-based PEC sensor displayed an improved “signal-on” photocathodic current response. The CdSe@SiO_2_ quantum dots, as a quenching agent, were introduced *via* a hybridization chain reaction to achieve a “signal off” photocurrent response, and then the PEC response switched to a “signal on” state after the addition of the detected species, Pb^2+^ (Fig. 6A). TAPP-COF is a p-type material that is beneficial to photogenerated electron–hole pair transfer.^139^ Hence, the as-prepared PEC detection sensor displayed a wide linear range from 0.05 nM to 100 nM with a LOD of 0.012 nM (Fig. 6C). Moreover, the flexibility of TAPP-COFs film was excellent, indicating its broad potential application in wearable PEC sensing devices (Fig. 6B).^140–145^

**Table 6:** Covalent organic framework-based photoelectrochemical monitoring of environmental pollutants^a^

Working electrode	Analyte	LOD	Linear concentration range	Ref.
TAPP-COF	Hg^2+^	0.012 nM	0.05 nM∼1000 nM	124
CoPc-PT-COF@Cu-MOF	Cr(iii)	14.5 fM	0.1 pM∼100 nM	126
F-COF/TiO_2_ NTAs	Dopamine	0.032 μM	0.1 μM∼300 μM	125
NH_2_-UiO-66/TpPa-1-COF	Dibutyl phthalate	30 pM	0.1 nM∼100 μM	127

a TAPP-COF: porphyrin-based covalent organic framework; CoPc-PT-COF: tetra-amine cobalt phthalocyanine-2,9-bis[*p*-(formyl) phenyl]-1,10-phenanthroline-covalent organic framework; F-COF: fluoro-substituted covalent organic frameworks; TpPa-1-COF: *p*-phenylenediamine-covalent organic framework.

Alternatively, the combination of COFs with other materials could enhance their performance in PEC detection platforms by providing more reactive sites for the determination of target environmental contaminants. In a study by Wang *et al.*, a heterostructure was formed between TiO_2_ NTAs and F-COFs through a simple hydrothermal method (Fig. 6D and E) and then used for constructing a PEC detection platform for dopamine.^125^ The introduction of F-COFs enhanced the visible light absorption and photogenerated electron–hole pairs separation efficiency. Meanwhile, the porous F-COFs processed a large specific surface area, and π–π interactions could be formed between aromatics of dopamine,^146^ which contributed to their ultra-sensitive detection activity. The resulting PEC sensor demonstrated excellent photocurrent response stability. More importantly, a LOD of 0.032 μM was obtained in an extended linear range (Fig. 6F).

Heterostructure COF/MOF hybrids have been fabricated as multifunctional materials for sensing applications.^127,147^ In 2021, Zhang *et al.* prepared a CoPc-PT-COF@Cu-MOF-based PEC-EC dual-mode biosensor for Cr(iii) quantification.^126^ The 2D CoPc-PT-COF was *in situ* grown on a Cu-MOF by covalent binding between the carboxyl group in the Cu-MOF and the amino group in the CoPc-PT-COF. The DNA strands could be easily anchored on the composite *via* strong interaction owing to its large specific surface area and high porosity. Furthermore, due to the specific recognition between DNA and Cr(iii), the composite-based biosensor could be used to trace Cr(iii). The photocurrent of composite (419 nA) was about 14.5 times greater than that of Cu-MOF, indicating that the heterojunction between Cu-MOF and CoPc-PT-COF enhanced the photoelectric conversion efficiency. So, the obtained composite-based PEC biosensor displayed a LOD of 14.5 fM within the Cr(iii) concentration range of 0.1 pM to 100 nM. Overall, porous COFs have significant potential for PEC sensing applications against environmental contaminants.

### 2.4. Graphitic carbon nitride

Graphitic carbon nitride (g-C_3_N_4_) is a well-known metal-free polymeric semiconductor that exhibits excellent visible light response and environmental friendliness.^148–151^ In recent years, g-C_3_N_4_ has been utilized in the area of PEC detection of environmental contaminants^120,152–156^ (Table 7). However, for bulk g-C_3_N_4_, the PEC detection performance is poor due to its relatively low photoelectric conversion efficiency. Activating pristine g-C_3_N_4_ with various treatments to achieve a porous structure is indeed an excellent way to speed up the separation of photogenerated charge pairs. Specifically, the methods employed to treat bulk g-C_3_N_4_ include chemical exfoliation and thermal oxidation.^149,157,158^

**Table 7:** Graphitic carbon nitride-based PEC sensing platform for tracing environmental pollutants^a^

Working electrode	Analyte	LOD	Linear concentration range	Ref.
A-CN	Cu^2+^	0.31 μM	0.2 μM∼50 μM	163
3DBC-C_3_N_4_	Cu^2+^	0.38 nM	1 nM∼100 nM	43
GA-C_3_N_4_	Cu^2+^	N/A	0.4 μM∼7.6 μM	164
F-g-C_3_N_4_	Cr(vi)	0.006 μg L^−1^	0.01 μg L^−1^∼1000 μg L^−1^	165
BiOI/CN_*x*_	Cr(vi)	0.1 μM	0.5 μM∼190 μM	166
g-C_3_N_4_@CdS QDs	Hg^2+^	12 nM	20 nM∼550 nM	167
g-C_3_N_4_/CB	Cd^2+^	2.1 nM	0∼700 nM	168
	Pb^2+^	0.26 nM	0∼300 nM	
	Hg^2+^	0.22 nM	0∼500 nM	
ND-g-CN	Ciprofloxacin	20 ng L^−1^	60 ng L^−1^∼19.1 μg L^−1^	169
CNNS-Cu	*p*-Nitrotoluene	0.13 μM	0.1 μM∼100 μM	170
Au/PCN-S	Oxytetracycline	0.34 nM	0.5 nM∼200 nM	162
Bi/BiVO_4_/g-C_3_N_4_	Oxytetracycline	0.0033 nM	0.01 nM∼1000 nM	171
Bi/CV-PCN	Enrofloxacin	3.3 fg mL^−1^	0.01 pg mL^−1^∼1 μg mL^−1^	172
Au-doped 3D CN	Chloramphenicol	0.1 pM	0.5 pM∼300 nM	173
α-Fe_2_O_3_/d-C_3_N_4_	Penbritin	0.0125 pM	0.5 pM∼50 nM	62
ZnPc/CN	Sulfadimethoxine	0.03 nM	0.1 nM∼300 nM	174
BiFeO_3_/utg-C_3_N_4_	Ampicillin	0.33 pM	1 pM∼1 μM	175
ZnIn_2_S_4_/g-C_3_N_4_	Bisphenol A	0.016 μM	0.05 μM∼30 μM	156
g-C_3_N_4_/BiOI	Bisphenol A	26 ng mL^−1^	80 ng mL^−1^∼3.2 μg mL^−1^	176
g-C_3_N_4_/CuO	Bisphenol A	6.2 ng L^−1^	0.02 ng L^−1^∼10 ng L^−1^	177
NiO-Ni-GCN	Octylphenol	3.3 nM	10 nM∼1 μM	178
g-C_3_N_4_/Bi_24_O_31_Cl_10_	Enrofloxacin	0.167 fM	0.5 fM∼100 fM	179
Bi/CV-PCN	Enrofloxacin	3.3 fg mL^−1^	0.01 pg mL^−1^∼1 μg mL^−1^	172
S-BN/Au/CN	Diazinon	6.8 pM	0.01 nM∼100 nM	180
CoN/g-C_3_N_4_	Atrazine	3.3 × 10^−5^ fM	1.0 × 10^−4^ fM∼10 fM	181
Ag_2_CrO_4_/g-C_3_N_4_/GO	Chloramphenicol	0.29 pM	0.5 pM∼50 nM	182
g-C_3_N_4_-AuNPs	Triclosan	0.601 pM	2 pM∼800 pM	183
C_3_N_4_-rGO	Rutin	1.78 nM	5 nM∼140 μM	184
CoO/Au/g-C_3_N_4_	Microcystin-LR	0.01 pM	0.1 pM∼10 nM	185
BiVO_4_/2D-C_3_N_4_	Microcystin-LR	0.042 pg L^−1^	0.5 pg L^−1^∼10 μg L^−1^	186

a A-CN: alkalized C_3_N_4_; 3DBC-C_3_N_4_: three-dimension branched crystalline carbon nitride; GA-C_3_N_4_: graphene-analogue carbon nitride; F-g-C_3_N_4_: formate anion-incorporated graphitic carbon nitride; Au/PCN-S: Au nanoparticle-decorated phosphorus-doped porous ultrathin C_3_N_4_ nanosheets; Au-doped 3D CN: Au nanoparticle doped three-dimensional graphitic carbon nitride; CNNS-Cu: copper cluster modified carbon nitride nanosheets; ZnPc/CN: zinc phthalocyanine/graphitic carbon nitride; utg-C_3_N_4_: ultrathin graphite-like carbon nitride; S-BN/Au/CN: sulfur doped hexagonal boron nitride/Au nanoparticles/graphitic carbon nitride; CB: carbon black; NS: nanosheets.

For the chemical exfoliation process, the morphology and porosity of the porous carbon nitride are determined by the choice of etchant and the reaction conditions.^159^ Common exfoliation agents include K_2_Cr_2_O_7_ + H_2_SO_4_, KMnO_4_ + H_2_SO_4_, and HNO_3_ + H_2_SO_4_.^160^ However, these chemical agents pose environmental and safety concerns due to their hazardous nature. Thermal oxidation is another method for producing porous carbon nitride. This process disrupts the hydrogen bonds in carbon nitride framework, leading to the formation of porous carbon nitride nanosheets with a large specific surface area and a thin sheet structure.^161^ However, those above two aforementioned approaches can harm the environment. Further, it requires high-quality bulk g-C_3_N_4_, and the process is complicated and time-consuming.

**Fig. 7:**
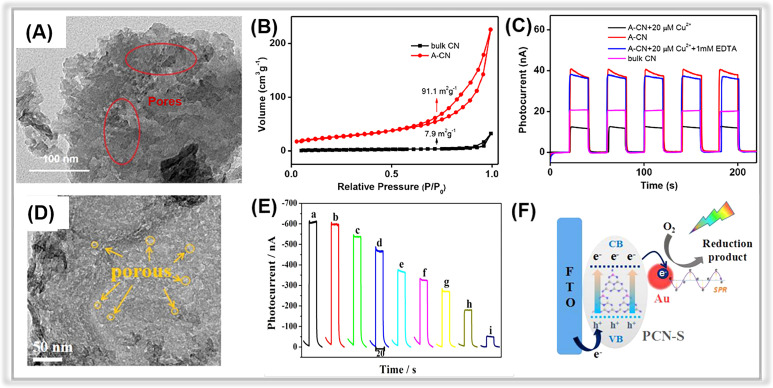
(A) TEM image of A-CN. (B) Nitrogen adsorption–desorption isotherm curves for bulk CN and A-CN. (C) Photocurrent responses of bulk CN and A-CN. Adopted from ref. 163. Copyright 2020, with permission of Elsevier. (D) TEM images of PCN-S. (E) Photocurrent responses of the Au/PCN-S at various oxytetracycline concentrations. (F) The photocurrent generation mechanism of Au/PCN-S under visible light irradiation. Adopted from ref. 162. Copyright 2018, with permission of Elsevier.

For instance, in a study by Peng and co-workers, phosphorus-doped porous carbon nitride nanosheets (PCN-S) were synthesized using element doping and thermal oxidation method.^162^ The PCN-S demonstrated an ultrathin nanosheet structure, a large specific surface area, and numerous surface pores (Fig. 7D). Moreover, the Au NPs with the LSPR effect were *in situ* decorated on the porous surface to yield an Au/PCN-S composite *via* a photo-reduction method. The composite was used to construct a self-powered PEC apta-sensor for the oxytetracycline (OTC) assay. Under visible light irradiation, electrons were excited to the CB of PCN-S and subsequently transferred to the Au NPs. They then reacted with dissolvable oxygen in the aqueous solution to produce superoxide oxygen species, which react with analyte-OTC (Fig. 7F). Owing to its strong visible light absorption ability and enhanced photogenerated charge pair separation efficiency, the Au/PCN-S composite-based PEC sensor demonstrated excellent performance in terms of a wide linear detection range (0.5∼200 nM), a low detection limit (0.34 nM) (Fig. 7E).

Different from traditional methods, alkaline hydrothermal treatment is a new effective approach to achieving porous carbon nitride nanosheets.^187,188^ In 2020, Liang *et al.* prepared a porous carbon nitride *via* an alkaline hydrothermal method.^163^ The hydroxide effectively exfoliated the 2D framework of the pristine bulk graphitic carbon nitride to obtain a porous structure with a large number of functional groups on its surface. The TEM image of activated g-C_3_N_4_ (A-CN) clearly revealed the presence of porous structures on its surface (Fig. 7A). Moreover, the specific surface area (SSA) of A-CN was about 10 times larger than that of bulk g-C_3_N_4_ (Fig. 7B), further confirming the existence of porous structures in A-CN. In Fig. 7C, the photocurrent response of A-CN was much higher than that of bulk g-C_3_N_4_ due to the porous structure and the large number of functional groups on the surface, which promoted photogenerated charge separation and transfer. Thus, the A-CN-based PEC sensor displayed an outstanding performance for Cu^2+^ detection with a low detection limit of 0.31 μM in the range from 0.2 μM to 50 μM.

Aiming to overcome the inevitable restacking and agglomeration of the bulk g-C_3_N_4_ nanosheets, the fabrication of three-dimensional (3D) porous g-C_3_N_4_ is another simple method. It not only prevents the agglomeration of nanosheets but also improves the utilization of irradiation through multiple reflections in its open framework.^189,190^ For instance, Zhang *et al.* developed a dual-photoelectrode for detecting chloramphenicol, employing Au NPs doping porous three-dimensional g-C_3_N_4_ and porous N-doped Cu_2_O/C used as the photoanode and photocathode, respectively.^173^ The small-scale Au NPs were uniformly distributed across the 3D CN nanosheets. The photocurrent of Au-doped 3D CN was much higher than that of bulk g-C_3_N_4_, and it also had the smallest electron transfer resistance. The porous 3D g-C_3_N_4_ improved both the visible light absorption ability and photoelectric conversion efficiency. Additionally, the presence of Au NPs with the LSPR effect provided extra energy for photogenerated charge carrier generation with the aid of hot electron transfer. As a result, the prepared apta-sensor exhibited an ultra-low detection limit (0.1 pM) and a wide linear detection range (0.5 pM∼300 nM).

### 2.5. MXene

MXene, a 2D transition metal carbide or nitride, has been widely used in various applications, including electromagnetic interference shielding, energy storage batteries, supercapacitors, and sensors.^38,191–194^ Porous MXenes have demonstrated outstanding chemical reactivity and hydrophilicity owing to their superior electrical conductivity and significant number of functional groups like –F and –OH, enabling them to form heterojunctions with semiconductors.^195^ Additionally, exposed connector metal sites, such as titanium on MXenes, present a higher redox reactivity compared to conventional carbon materials, positioning MXene nanolayers as ideal optoelectronic platforms. Recent reports on MXene-based PEC sensors for environmental pollutant detection are summarized in Table 8. These reports illustrate that MXene can be combined with a variety of materials, including graphene oxide, graphitic carbon nitride, metal oxide, metal halide, and metal sulfide, to construct composite hybrid photoelectrochemical detection platforms.

**Table 8:** MXene-based PEC sensors for environmental pollutant detection^a^

Working electrode	Analyte	LOD	Linear concentration range	Ref.
Ti_3_C_2_ MXene/SnS_2_	Cr(vi)	0.51 pM	1.0 pM∼0.1 mM	199
Ti_3_C_2_ MXene/BiVO_4_	Hg^2+^	1 pM	1 pM∼2 nM	200
CdS/Ti_3_C_2_ MXene	Cu^2+^	0.05 nM	0.1 nM∼10 μM	201
BiOBi_*x*_I_1−*x*_/Ti_3_C_2_ MXene	Hg^2+^	42.1 pM	0.1 nM∼1000 nM	202
BiOI/Ti_3_C_2_ MXene	l-cysteine	0.005 nM	0.01 nM∼10 μM	203
TiO_2(001)_/Ti_3_C_2_ MXene	Dopamine	0.52 μM	1.0 μM∼1000 μM	204
BiVO_4_/Ti_3_C_2_ MXene	Oxytetracycline	0.03 nM	0.1 nM∼100 nM	205
g-C_3_N_4_/Ti_3_C_2_ MXene	Ciprofloxacin	0.13 nM	0.4 nM∼1000 nM	196
Ti_3_C_2_ MXene/Bi_4_VO_8_Br/TiO_2_	Ciprofloxacin	0.3 nM	1 nM∼1500 nM	206
AgBr/Ti_3_C_2_ MXene	Chlorpyrifos	0.33 pg L^−1^	0.001 pg L^−1^∼1 ng L^−1^	207
Bi_4_VO_8_Br/Ti_3_C_2_ MXene	Streptomycin	0.3 nM	1 nM∼1000 nM	208
Au@PtAg/TiO_2_-Ti_3_C_2_ MXene	Ochratoxin A	1.73 fg mL^−1^	5 fg mL^−1^∼10 ng mL^−1^	209
Anode: TiO_2_/S-Ti_3_C_2_ MXene	Microcystin-RR	0.034 fM	0.1 fM∼1 nM	210
Cathode: MoS_2_/S-Ti_3_C_2_ MXene	Aflatoxin B1	0.73 pg mL^−1^	0.01 pg mL^−1^∼1 μg mL^−1^	211
MoS_2_-Ti_3_C_2_T_*x*_ MXene				
AgI/Ti_3_C_2_ MXene/GO	Sulfide	1.54 nM	5 nM∼200 μM	198

a TiO_2(001)_: active surface (001 facet) TiO_2_; AgI/Ti_3_C_2_ MXene/GO: AgI/3D porous Ti_3_C_2_ MXene/graphene aerogel.

**Fig. 8:**
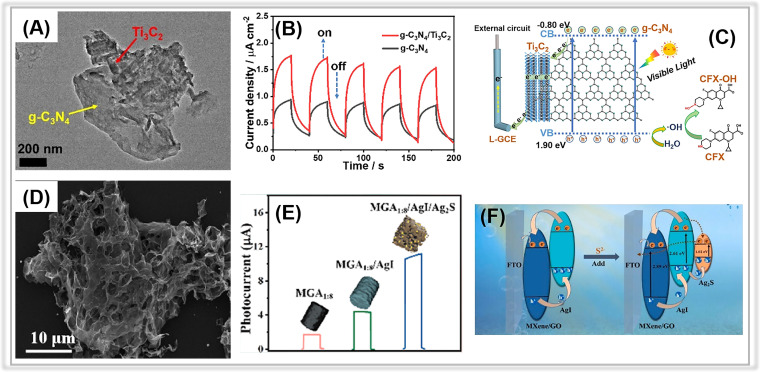
(A) TEM image of the g-C_3_N_4_/Ti_3_C_2_ MXene composite. (B) The transient photocurrents of g-C_3_N_4_ and g-C_3_N_4_/Ti_3_C_2_ MXene composite. (C) The proposed PEC mechanism at the g-C_3_N_4_/Ti_3_C_2_ MXene composite. Adopted from ref. 196. Copyright 2021, with permission of Elsevier. (D) SEM image of AgI/Ti_3_C_2_ MXene/GO composite. (E) The photocurrent responses of Ti_3_C_2_ MXene/GO, AgI/Ti_3_C_2_ MXene/GO, and Ag_2_S/AgI/Ti_3_C_2_ MXene/GO composites. (F) The possible electron-transfer mechanism of the AgI/Ti_3_C_2_ MXene/GO composite. Adopted from ref. 198. Copyright 2023, with permission of Elsevier.

Graphene oxide and graphitic carbon nitride can be effectively coupled with the porous MXenes due to the strong π–π interaction between them.^196,197^ For instance, in a study by Yuan and co-workers, the g-C_3_N_4_/Ti_3_C_2_ MXene composite was synthesized and proposed as a PEC sensing material for ciprofloxacin detection.^196^ In Fig. 8A, porous Ti_3_C_2_ MXene was observed on the surface of g-C_3_N_4_, suggesting a close connection between the two components facilitated by electrostatic self-assembly. Transient photocurrent measurements (Fig. 8B) revealed a photocurrent density of 1.36 μA cm^−2^ for the g-C_3_N_4_/Ti_3_C_2_ MXene, nearly twice that of g-C_3_N_4_. Meanwhile, the EIS of g-C_3_N_4_/Ti_3_C_2_ MXene has a smaller arc size compared to that of pure g-C_3_N_4_. These findings suggested that the introduction of Ti_3_C_2_ MXene promotes photogenerated charge transfer. The fabricated PEC sensor demonstrated an ultra-low detection limit of 0.13 nM over a broad linear range from 0.4 nM to 1000 nM.

These results imply that the tight interaction between Ti_3_C_2_ MXene and g-C_3_N_4_ facilitates the smooth transfer of photogenerated electrons from g-C_3_N_4_ to Ti_3_C_2_ MXene, whose Fermi level (0.58 V *vs.* NHE) is much more positive than the conduction band energy of g-C_3_N_4_ (Fig. 8C).

Similarly, another typical carbon material, graphene oxide, has also been coupled with porous Ti_3_C_2_ MXene to construct smart PEC sensing platforms. In 2023, Zhang *et al.* reported a “signal-on” PEC sensor for sulfide detection based on *in situ* growth of AgI on a 3D porous Ti_3_C_2_ MXene/graphene aerogel (AgI/Ti_3_C_2_ MXene/GO).^198^ The porous Ti_3_C_2_ MXene/GO was synthesized through the solvothermal method, followed by *in situ* decoration of AgI NPs onto the Ti_3_C_2_ MXene/GO. Fig. 8D depicts the typical three-dimensional interconnected skeleton structure of aerogel. The porous structure of Ti_3_C_2_ MXene/GO aerogel with large specific surface area favored the anchoring of AgI NPs. Upon the addition of detection analyte-sulfide (S^2−^), Ag_2_S formed on the surface of AgI/Ti_3_C_2_ MXene/GO, resulting in an enhanced photocurrent response due to the newly created Ag_2_S/AgI heterojunction (Fig. 8F). As a result, the associated PEC sensor revealed an outstanding S^2−^ detection activity, including a wide linear range (5 nM∼200 μM) and an ultra-low detection limit (1.54 nM).

Interestingly, MXene can serve as a co-catalyst to enhance the separation efficiency of photogenerated electron–hole pair.^204,205,212,213^ Recently, porous MXenes have been combined with various semiconductors (*e.g.*, TiO_2_, BiVO_4_, MoS_2_, and BiOI) to fabricate composite-based PEC platforms for environmental contaminant assays. For example, Ling and co-workers reported a PEC sensing platform based on a BiVO_4_/Ti_3_C_2_ MXene composite for oxytetracycline (OTC) detection.^205^ Within the composite, the small porous MXene nanosheets acted as a co-catalyst to promote photo-generated charge pair separation. This was confirmed by the enhanced photocurrent responses, weaker PL intensity, and smaller arc resistance values of BiVO_4_/Ti_3_C_2_ MXene. The introduction of MXene created a Schottky barrier between BiVO_4_ and MXene, effectively suppressing electron reflux. During the PEC detection process, photogenerated electrons in the CB of BiVO_4_ were transferred to MXene *via* the Schottky junction and then injected into a conductive substrate (in this case, ITO) to generate photocurrent. Simultaneously, holes in the VB of BiVO_4_ contributed to the current generation by oxidizing OTC molecules on the composite surface. Thus, the improved photocurrent resulted in a wide detection range (0.1∼100 nM), an ultra-low detection limit (0.03 nM), and excellent sensitivity for the corresponding PEC sensing platform.

Schottky heterojunctions have also been observed in other Ti_3_C_2_ MXene-containing composites. For instance, Ye *et al.* reported the utilization of a flower-like BiOI/2D Ti_3_C_2_ MXene (BiOI/Ti_3_C_2_) heterostructure composite as a photocathode for l-cysteine (L-Cys) PEC biosensing.^203^ The Schottky interaction between the two components enhanced charge transfer, resulting in a superior cathodic photocurrent signal. Similarly, a Schottky junction was also found in CdS nanoparticles and Ti_3_C_2_ MXene in other studies.^201^ The CdS/Ti_3_C_2_ heterostructure-based PEC sensing platform showed a linear response for Cu^2+^ ranging from 0.1 nM to 10 μM with a LOD of 0.05 nM, attributed to significantly improved charge carrier transfer at the CdS/Ti_3_C_2_ interface. In another report, Du *et al.* employed a wet-chemical method to fabricate AgBr/Ti_3_C_2_ Schottky heterojunction composites for self-powered PEC sensing of chlorpyrifos (CPF).^207^ The synergistic effect of the Schottky heterojunction between AgBr and Ti_3_C_2_ MXene, combined with metal–ligand charge transfer (MLCT), promoted photogenerated charge carrier separation and transfer. The PEC sensor demonstrated excellent performance in CPF monitoring, with a linear detection range of 0.001∼1 ng L^−1^ and a LOD of 0.33 pg L^−1^. Recently, porous MBenes, which are respective transition metal borides,^214–218^ have been applied in the areas of energy storage and electrocatalysis. Until now, there have been few reports on the application of MBenes to photoelectrochemical sensors. However, we believe that MBenes have great potential in the fabrication of PEC detection platforms.

### 2.6. Perspectives

In summary, over the past decade, metal oxides, MOFs, COFs, graphitic carbon nitride, and MXene have made significant advances in the construction of PEC analytical platforms for environmental contaminants. Due to their porous structure, large specific surface area and abundant active sites, PEC sensors based on photoactive porous material exhibit superior optoelectronic response. (1) Among the photoactive metal oxides, TiO_2_ nanotubes or nanoarrays with unique porous channels can efficiently transmit photogenerated charge pairs. PEC sensors based on TiO_2_ nanotubes or nanoarrays can be applied to detect heavy metal ions and organic pollutants, such as Cr(vi) and atrazine. (2) MOFs and COFs both exhibit excellent photoelectrochemical detection performance against organic pollutants. Moreover, derivatives of MOFs have demonstrated robust PEC detection activities against environmental toxic species. (3) Bulk g-C_3_N_4_ can be treated with a variety of methods to obtain a porous structure, including chemical exfoliation, thermal oxidation, and alkaline hydrothermal treatment. Meanwhile, the construction of 3D frameworks is another approach to obtain porous structures. A number of research works have demonstrated that porous graphitic carbon nitride-based PEC sensors exhibit outstanding detection activity in terms of ultra-low detection limit and wide linear detection range. (4) Porous MXenes characterized by high redox reactivity, superior electrical conductivity, and rich functional groups, can effectively be coupled with other materials to construct composite hybrid PEC platforms for pollutant determination. Therefore, porous materials have great potential for the fabrication of PEC sensors.

## 3. Applications for determining the environmental pollutants

### 3.1. Heavy metal ions

Heavy metal ions are highly detrimental environmental contaminants that can enter living organisms through the food chain and cause enzyme inhibition, poor antioxidant metabolism, DNA damage, and depletion of protein sulfhydryl groups.^219–221^ Copper (Cu), iron (Fe), manganese (Mn), cobalt (Co), and zinc (Zn) are required for biological functions at relatively low concentrations but become toxic in excessive amounts. Low amounts of lead (Pb), mercury (Hg), chromium (Cr), cadmium (Cd), and arsenic (As) are nevertheless dangerous.^222,223^ The World Health Organization (WHO) has established stringent restrictions regarding the concentration of heavy metal ions in drinking water. In recent years, many efforts have been dedicated to developing porous material-based PEC sensors for monitoring heavy metal ions.^46,167,224,225^ In this section, we present a selection of relevant works on PEC detection of heavy metal ions.

#### 3.1.1. Cr(vi)

Hexavalent chromium (Cr(vi)) has been identified as one of the most toxic heavy metal ions, posing a significant threat to biological and ecological systems.^226^ Thus, it is critical for detecting Cr(vi) in water bodies. Several studies have been conducted to improve the PEC detection performance for Cr(vi).^227^ For instance, Qiao *et al.* constructed a NiCo-LDHs-modified TiO_2_ NTAs/Ti photoelectrode using anodization and electrodeposition techniques.^55^ The proposed PEC sensor demonstrated a linear relationship between photocurrents and Cr(vi) concentrations ranging from 10 μM to 400 μM, with a LOD of 3.2 μM. Moreover, the sensor displayed no discernible reactions even when subjected to various interfering ions at high concentrations (*e.g.*, Cr^3+^, Sn^4+^, Cd^2+^, Pb^2+^, Mn^2+^, Na^+^, and Fe^2+^), indicating its strong selectivity for Cr(vi). More importantly, the practical application of the developed PEC sensor was examined by detecting Cr(vi) in river water, demonstrating its excellent potential in real-world scenarios. Additionally, porous graphitic carbon nitride has been employed for the construction of PEC platform for Cr(vi) detection. For instance, Fang *et al.* designed a formate anion-incorporated graphitic carbon nitride (F-g-C_3_N_4_)-based PEC sensor for determining trace amounts of Cr(vi).^165^ The porous F-g-C_3_N_4_ demonstrated enhanced photogenerated charge separation efficiency compared with bulk g-C_3_N_4_. Furthermore, when combined with the molecularly imprinted polymers (MIPs), F-g-C_3_N_4_ served as a sensing platform for Cr(vi) determination. The resulting sensor exhibited remarkable sensitive, demonstrating a linear range from 0.01 μg L^−1^ to 100 μg L^−1^, and achieving a LOD of approximately 0.006 μg L^−1^.

The construction of heterojunctions was another strategy to improve the PEC detection activity for Cr(vi). For instance, in 2021, Cheng *et al.* devised a quick and highly sensitive PEC sensor for determining Cr(vi) in water samples.^166^ The photoactive electrode consisted of a p–n BiOI/CN heterojunction composite. The CN nanosheets were effectively coupled to the BiOI with a needle-like petal structure. The photocurrent density of BiOI/CN was significantly higher than that of pure CN and BiOI, suggesting that the introduction of porous CN enhanced the visible light absorption. Moreover, the BiOI/CN processed the smallest charge transfer resistance due to its unique structure, leading to increased active area and electric conductivity of the photoelectrode. Furthermore, the PEC detection performance of the composite-based electrode for Cr(vi) was studied at various Cr(vi) concentrations. It displayed a wide linear detection range of 0.5∼190 μM with a LOD of 0.1 μM. Under visible light irradiation, the electrons in the VB of CN and BiOI were excited to their CB. Due to the Fermi level of the p-type BiOI being close to the VB and that of the n-type CN being close to the CB, electrons in the CB of the CN tended to diffuse into the BiOI, while holes in the VB were transferred from the BiOI to the CN. The electrons in CB of CN could react with Cr(vi) to convert it to harmless Cr(iii). As a result, an interfacial electric field was formed between BiOI and CN, facilitating the separation of photogenerated charge pairs.

#### 3.1.2. Hg^2+^

**Fig. 9:**
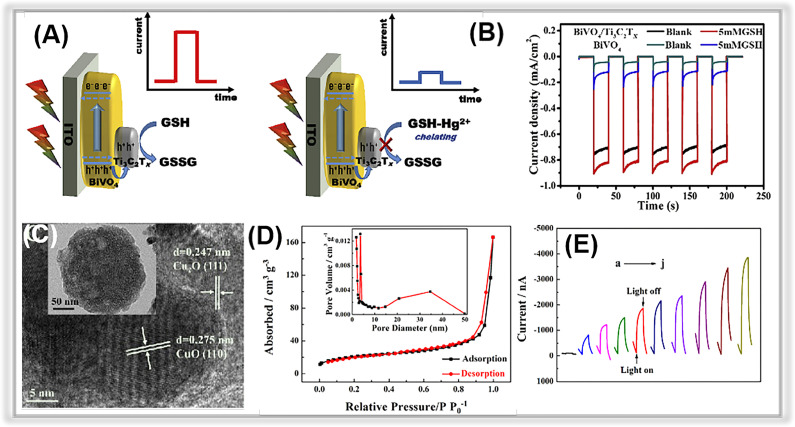
(A) The mechanism of sensing Hg^2+^ with the photoelectrochemical method. (B) The photocurrent responses of BiVO_4_ and Ti_3_C_2_ MXene/BiVO_4_. Adopted from ref. 200. Copyright 2020, with permission of Elsevier. (C) TEM and HR-TEM images of Cu_2_O–CuO–TiO_2_ composites. (D) Nitrogen adsorption–desorption isotherms of the Cu_2_O–CuO–TiO_2_. (E) Photocurrent responses with different Pb^2+^ concentrations. Adopted from.^112^ Copyright 2022, with permission of Elsevier.

Another harmful heavy metal contaminant commonly found in water bodies is Hg^2+^. It can cause damage to the brain, kidneys, and central nervous system. EPA has defined a maximum allowable limit of 10 nM of inorganic mercury in drinking water.^228,229^ Therefore, it is critical to detect it accurately. Porous MXene-based composites have been employed to construct PEC platforms for monitoring Hg^2+^ in water bodies. For instance, Xiao *et al.* prepared BiOBi_*x*_I_1−*x*_/Ti_3_C_2_ MXene Schottky heterojunction nanocomposites using an electrostatic self-assembly method.^202^ Porous Ti_3_C_2_ MXene possesses a high specific surface area, excellent electrical conductivity, and an abundance of surface chemical groups. In addition, the Schottky heterojunction of Ti_3_C_2_ MXene and BiOBi_*x*_I_1−*x*_ significantly enhances the separation rate of photogenerated charge pairs, resulting in a higher photocurrent signal compared to BiOBi_*x*_I_1−*x*_ alone. During the detection of Hg^2+^, an ultra-low detection limit of around 42.1 pM was obtained, ranging from 0.1 nM to 1000 nM. Moreover, this sensor has been successfully employed to determine Hg^2+^ in environmental samples. Similarly, Jiang *et al.* used a BiVO_4_/Ti_3_C_2_ MXene composite to construct a visible light-driven PEC sensor for probing Hg^2+^.^200^ A single Ti_3_C_2_ MXene layer was coated on the BiVO_4_ film. Electrochemical impendence spectroscopy measurements revealed a smaller arc diameter for the interfacial charge transfer resistance of the composite photoelectrode compared to pure BiVO_4_. Further, the photocurrent signal of the composite-based electrode was higher than that of BiVO_4_ (Fig. 9B). Meanwhile, the photocurrents of BiVO_4_ and BiVO_4_/Ti_3_C_2_ MXene-based electrodes increased upon the addition of glutathione (GSH) (Fig. 9A). The porous MXene, a metal-like material with high conductivity, was coupled with BiVO_4_ to form a Schottky junction, facilitating charge-pair separation. Thus, the performance of the PEC analysis for Hg^2+^ demonstrated a detection limit of 1.0 pM within a range of 1 pM to 2 nM.

#### 3.1.3. Cu^2+^

Cu^2+^ is required by all living organisms. However, excessive amounts of copper can cause hepatic or renal damage, as well as gastrointestinal disturbances. Therefore, it is critical to detect Cu^2+^ concentration levels in environmental and biological samples.^230,231^ Porous material-based PEC sensors for monitoring Cu^2+^ have been investigated by several researchers.^43,73,163^ For instance, in a study by Xu and co-workers, a porous graphene-analogue carbon nitride (GA-C_3_N_4_) was derived from the graphitic C_3_N_4_.^164^ GA-C_3_N_4_ is an ideal candidate for a PEC sensor to determine Cu^2+^ due to its thin layer structure and high specific surface area. The fabricated sensor demonstrated a linear photocurrent response with varying Cu^2+^ concentrations over a wide range of 0.4∼7.6 μM.

#### 3.1.4. Pb^2+^

Even at low concentrations, lead ions (Pb^2+^) represent a major threat to both species and human health. The United States Environmental Protection Agency (EPA) has established that the maximum allowed quantity of Pb^2+^ ions in drinking water is 15 ppb (72 nM),^232^ hence, reliable detection is critical.^233–235^ For example, in a recent study by Yu and co-workers, a label-free and ultrasensitive PEC biosensor was developed for the determination of Pb^2+^.^112^ The NH_2_-MIL-125(Ti) was synthesized *via* a hydrothermal method and subsequently doped with Cu. The Cu^2+^-doped-titanium-based metal–organic framework (Cu^2+^/NH_2_-MIL-125(Ti)) was calcinated to create a porous Cu_2_O–CuO–TiO_2_ heterojunction composite. In Fig. 9C, the composite exhibited a disk-like morphology, and the presence of Cu_2_O and CuO was confirmed by HR-TEM measurements. The nitrogen adsorption–desorption isotherm of the composite was shown in Fig. 9D, revealing a specific surface area (SSA) of up to 75.1 m^2^ g^−1^ and an average pore size of 18.4 nm. Consequently, the porous structure and high SSA were beneficial for enhancing light absorption and facilitating the photogenerated charge transport during PEC detection. In Fig. 9E, the composite-based sensor exhibited a broad detection range of 10 fM to 1 μM and a low detection limit (6.8 fM).

#### 3.1.5. Perspectives

In summary, significant efforts have been devoted to the fabrication of photoelectrochemical sensing platforms based on photoactive porous materials for heavy metal ion assays. These platforms exhibited improved sensing activity, such as excellent sensitivity, an ultra-low detection limit (pM), and a wide linear range. The advantage of porous materials in the detection of heavy metal ions has been proven by the reports cited above: (1) porous material with a large surface area and abundant functional groups facilitates interaction with recognition elements or heavy metal ions; (2) fast photogenerated charge carrier separation and transfer rate within the photoactive porous materials. However, some challenges still remain with the porous material-based PEC detection sensors for heavy metal ion assays and need to be resolved: (1) some toxic heavy metal ions, such as arsenic (As^5+^), have not yet been monitored by porous materials-based PEC sensors; (2) despite the great potential of metal–organic frameworks for PEC detection of heavy metal ions, few works have been reported; and (3) the stability, repeatability, multiple detection, and anti-interference of heavy metal ion PEC sensors still require enhancement.

### 3.2. Organic pollutants

Organic pollutants found in wastewater encompass a wide range of compounds, including phenolics, antibiotics, pesticides, and toxins. These substances are extremely toxic and pose serious risks to human health. Thus, developing environmentally friendly methods for accurately determining these contaminants has become a focus of worldwide.^236,237^

#### 3.2.1. Phenolics

Phenols and their derivatives are known for their recalcitrance and acute toxicity. Under EPA regulations, the permissible limit for phenol in surface water is no more than 1 ppb. High concentrations of phenolic compounds can have adverse effects on human health, such as salivation and anorexia.^238^ Hence, increasing attention has been focused on detecting these phenolic species.^176^ Porous carbon nitride is an excellent choice for developing PEC platforms for phenolic assays. For instance, Chen *et al.* produced a sensitive PEC sensor for *p*-nitrotoluene (*p*-NT) detection based on a copper cluster-modified porous carbon nitride nanosheet composite (CNNS-Cu).^170^ Under light irradiation, the Cu^2+^ cluster captures the electrons on the CB and converts them to Cu^+^. Meanwhile, the electrons excited from the VB of the CNNS are occupied by Cu^2+^, which enhances the separation and migration of photogenerated charge pairs. Upon the introduction of *p*-NT, Cu^2+^ facilitates *p*-NT reduction *via* Cu^2+^/Cu^+^ redox reduction. As a result, this PEC sensor exhibits a wide linear detection range (0.1∼100 μM) and a low detection limit of 0.13 μM. In addition to graphitic carbon nitride with a porous structure, TiO_2_ nanotubes with unique pore channels have been employed in PEC applications for phenols determination. For example, Wang *et al.* developed a novel PEC sensor for bisphenol A detection by combining a TiO_2_ nanotubes/CdS heterostructure with inorganic framework molecular technology in PEC sensor analysis.^78^ The porous pore structures of TiO_2_ nanotubes favor photogenerated charge transfer, and the formation of CdS/TiO_2_ heterojunctions enhances the visible light absorption and improves photoinduced charge pair separation efficiency. Moreover, the inorganic framework molecular imprinting method creates plenty of recognition sites for target analytes (BPA). Hence, the fabricated sensor demonstrated excellent PEC sensing performance (detection range: 1∼100 pM, low limit detection: 0.5 pM).

#### 3.2.2. Antibiotics

**Fig. 10:**
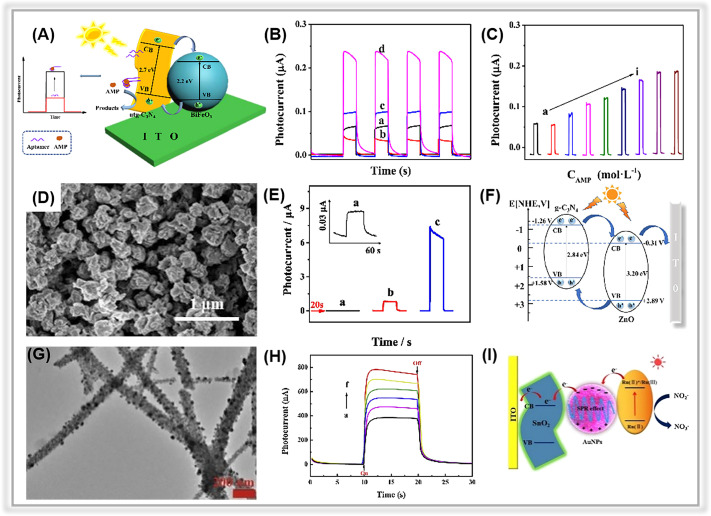
(A) The illustration of the PEC ampicillin apta-sensor based on the BiFeO_3_/utg-C_3_N_4_ composite. (B) The photocurrent signals of (a) utg-C_3_N_4_, (b) BiFeO_3_, (c) BiFeO_3_/bulk-C_3_N_4_, and (d) BiFeO_3_/utg-C_3_N_4_. (C) Photocurrent responses of the as-prepared apta-sensor at various ampicillin concentrations. Adopted from ref. 175. Copyright 2019, with permission of Elsevier. (D) SEM image of the ZnO/g-C_3_N_4_ composite. (E) The transient photocurrent responses of (a) g-C_3_N_4_, (b) ZnO, and (c) ZnO/g-C_3_N_4_. (F) Electron transfer mechanism for ZnO/g-C_3_N_4_. Adopted from ref. 117. Copyright 2023, with permission of Elsevier. (G) TEM images of SnO_2_-Au NPs. (H) The photocurrent responses of SnO_2_-Au NPs with different concentrations of nitrite. (I) The possible mechanism of PEC sensing nitrite. Adopted from ref. 85. Copyright 2020, with permission of Elsevier.

Antibiotics are widely used in human healthcare, aquaculture, and crop growth. However, the misuse and discharge of antibiotics into the environment have raised significant concerns.^239^ The release of antibiotic-containing wastewater poses a severe threat to both human health and ecological systems.^14,240–242^ Consequently, there is an urgent need to develop sensitive and effective methods for antibiotics detection.^243–246^ Recently, Liu *et al.* reported a simple PEC apta-sensor for tracing sulfadimethoxine (SDM) based on a zinc phthalocyanine/graphitic carbon nitride composite (ZnPc/CN).^174^ By modifying porous graphitic carbon nitride nanosheets with visible/near-infrared light-responsive ZnPc, ZnPc/CN nanocomposites were created. The porous CN nanosheets not only provided abundant sites for binding ZnPc, but also facilitated transport of photogenerated charges. The assembled PEC apta-sensor exhibited a linear correlation with SDM concentration in the range of 0.1∼300 nM, with a LOD of 0.03 nM. Further, it demonstrated excellent selectivity towards SDM even in the presence of familiar interferences (*e.g.*, oxytetracycline, kanamycin, bisphenol A, and diclofenac). Similarly, using the same sensing strategy,^175^ a facile PEC apta-sensor based on a p-type BiFeO_3_/n-type porous ultrathin graphitic carbon nitride (BiFeO_3_/utg-C_3_N_4_) was constructed (Fig. 10A). The BiFeO_3_/utg-C_3_N_4_ composite was obtained by a simple electrostatic interaction method. The TEM image confirmed the coupling of BiFeO_3_ NPs to the porous graphitic carbon nitride surface. In Fig. 10B, the composite-based electrode exhibited a significantly higher photocurrent intensity than pure BiFeO_3_ and utg-C_3_N_4_, indicating the suppression of photogenerated charge pair recombination due to the presence of p–n heterojunction in the composite. The EIS analysis further confirmed the efficient charge separation and transfer in the BiFeO_3_/utg-C_3_N_4_ composite-based electrodes, as they exhibited the smallest charge transfer resistance among all the electrodes. The fabricated PEC sensor was applied to ampicillin detection. As displayed in Fig. 10C, the apta-sensor achieved a relatively low LOD of 0.33 pM within a wide range from 1 pM to 1 μM.

Further, the porous graphitic carbon nitride can be combined with metal oxides derived from metal–organic frameworks to construct heterojunction composites. In 2023, Jiang *et al.* synthesized a MOF-derived ZnO nanopolyhedra/g-C_3_N_4_ composite *via* calcination of ZIF-8 and melamine.^117^ The g-C_3_N_4_ nanosheets were covered on the ZnO surface (Fig. 10D). In Fig. 10E, the remarkable photocurrent of the ZnO/g-C_3_N_4_ composite-based electrodes compared to pure ZnO was attributed to the presence of heterojunctions that facilitate the separation of photogenerated electron–hole pairs. A plausible electron transfer mechanism was proposed in Fig. 10F. Due to the suitable band energy levels of ZnO and g-C_3_N_4_, a type-II heterojunction is formed at the contact interface of the two components. As a result, the corresponding PEC apta-sensor demonstrated an ultra-low LOD (1.49 pM) in a wide linear range spanning from 5 pM to 200 nM. Furthermore, in addition to aptamer-based PEC sensors, recognition element-free sensors have been employed for the detection of antibiotics. For instance, Yan *et al.* presented a non-recognition element PEC sensor using a porous nitrogen-deficient graphitic carbon nitride nanosheet (ND-g-CN) for ciprofloxacin (CIP) detection.^169^ Notably, the presence of nitrogen vacancies serves as traps, effectively suppressing charge recombination, while the porous sheet structure promotes the separation and transfer of photogenerated charge carriers. Thus, the PEC sensor realized an ultra-sensitive determination of CIP (LOD: 20 ng L^−1^).

#### 3.2.3. Pesticides

Pesticides, even in low concentrations, can have a significant impact on the ecosystem and human health due to their high toxicity and resistance to decomposition.^247^ Therefore, extensive efforts have been made to trace these pesticides in the environment.^46,115,248,249^ In 2020, Qin *et al.* synthesized CdS nanocrystal-functionalized porous ultrathin MnO_2_ nanosheet composites (CdS/MnO_2_).^77^ They established a linear relationship between the concentration of organophosphorus pesticides (OPs) and photocurrent by utilizing enzymatic etching of MnO_2_ nanosheets and enzyme inhibition by OPs. The constructed PEC sensor had the merits of excellent stability, a wide linear range (0.05∼10 ng mL^−1^), and outstanding sensitivity.

Different from the enzyme-based biosensor, a self-powered PEC apta-sensor was constructed for diazinon (DZN) detection based on porous graphitic carbon nitride (CN).^180^ In this PEC apta-sensor, sulfur-doped h-BN (S-BN) was combined with the porous graphitic carbon nitride (CN), and Au nanoparticles with localized surface plasmon resonance (LSPR) effect acted as bridges to create a Z-scheme heterojunction, thereby enhancing the separation efficiency of photogenerated charge pairs. The proposed PEC sensor exhibited the benefits of a low detection limit (6.8 pM), a broad linear detection range (0.01∼10 000 nM), and excellent selectivity for DZN detection.

#### 3.2.4. Toxins

Toxins are harmful compounds produced by living cells or organisms, including microcystin-LR (MC-LR), ochratoxin A (OTA), and aflatoxin B1 (AFB1).^250^ Therefore, the development of simple and sensitive methods for detecting such toxins is critical. Recently, Li *et al.* constructed a photoelectrochemical sensor for MC-LR detection, employing a bismuth/two-dimensional graphitic carbon nitride/deoxyribonucleic acid (BiVO_4_/2D-C_3_N_4_/DNA) aptamer system.^186^ The porous graphitic carbon nitride nanosheets were modified onto the surface of the BiVO_4_ film photoelectrode. The coupling of 2D-C_3_N_4_ with BiVO_4_ formed a type-II heterojunction, enhancing the transfer capacity of photogenerated electron–hole *via* an internal electric field effect. Additionally, 2D-C_3_N_4_ effectively absorbed the DNA aptamer probe owing to its large area of π–π bonds. Thus, the sensor achieved a LOD of 0.04 pg L^−1^ within the range of 0.5 pg L^−1^∼10 μg L^−1^. Apart from the conventional type-II heterojunction, the porous g-C_3_N_4_ has been combined with various semiconductors and electron transfer mediators (*e.g.*, Ag, Au, and Pt) to construct Z-scheme heterojunction composites. For instance, Tang *et al.* developed a self-powered photoelectrochemical apta-sensor for MC-LR determination based on a Z-scheme CoO/Au/g-C_3_N_4_ heterojunction composite.^185^ In this composite, both CoO and Au nanoparticles were well anchored to the surface of the porous g-C_3_N_4_. The well-matched energy bands between CoO and g-C_3_N_4_, along with the presence of Au NPs as electron transfer mediators, significantly improved the separation efficiency of photogenerated charge pairs. Thus, the detection limit for this sensor was lowered to 0.01 pM over a wide range from 0.1 pM to 10 nM.

#### 3.2.5. Perspectives

Overall, PEC sensors based on photoactive porous materials hold significant promise for the sensitive and selective detection of organic environmental pollutants. Metal oxides (such as TiO_2_), metal–organic frameworks, covalent organic frameworks, graphitic carbon nitride, and MXene all exhibit huge potential for detecting various types of toxic organic contaminants, including phenolics, antibiotics, pesticides, toxins, and dyes. These materials demonstrate robust photoelectrochemical detection performance with high sensitivity and excellent selectivity. Nevertheless, there are still some challenges in this field: (1) most of reported PEC sensors for organic environmental pollutants assays still rely on the target as the electron donor during the detection procedures, which results in relatively low interference in complex samples. Therefore, there is a need for the development of novel photoactive porous materials that can directly interact with organic analytes. (2) The anti-fouling properties of photoactive porous materials need improvement due to the adsorption of organic molecules and their derivatives on the surface of these materials.

### 3.3. Non-metal ion pollutants

Apart from heavy metal ions and organic pollutants, there is growing concern regarding non-metal ion pollutants, including nitrites, sulfides, and cyanides.^251^ Recently, several works have focused on the PEC detection of these non-metal ion pollutants in the environment.^252,253^ In 2017, Su *et al.* described a novel PEC sensor for sulfide detection using Cu_2_O-decorated TiO_2_ nanotubes.^84^ The p–n heterojunction in the porous Cu_2_O–TiO_2_ heterostructure effectively suppressed the recombination of photogenerated charge pairs, leading to enhanced photocurrent response. The detection of sulfide by this sensor displayed a wide linear range from 1 μM to 300 μM, and the LOD was 0.6 μM. Furthermore, it has been successfully employed for accurate sulfide monitoring in tap and lake water, achieving outstanding recoveries ranging from 99.2% to 103%.

In addition to the detection of toxic sulfides in water bodies, the PEC sensing platform has also been utilized for tracing nitrides. For instance, Luo *et al.* constructed a porous three-dimensional (3D) network of SnO_2_ nanofibers on an ITO substrate using an electrospinning technique, followed by the electrodeposition of gold nanoparticles (Au NPs).^85^ The presence of porous SnO_2_ nanofibers with a 3D network structure was beneficial for the photogenerated charge pair separation and transfer (as shown in Fig. 10G). Moreover, Au NPs with the LSPR effect suppressed recombination of electron–hole pairs. By utilizing the photosensitizer-Ru(bpy)_3_^2−^, the photocurrent response was further increased. As illustrated in Fig. 10I, under the 473 nm light irradiation, the SnO_2_ could not be excited, whereas Au NPs and Ru(bpy)_3_^2−^ were both excited. Ru^2+^ was excited into unstable Ru^2+^*, and the electrons were transferred to the Au or SnO_2_ nanofibers. These electrons were then transferred into the conduction band of SnO_2_ with the aid of Au NPs in the surface plasma state. The Ru^2+^ ions were converted to Ru^3+^ ions, which then reacted with NO_2_^−^ to form NO_3_^−^ and Ru^2+^ ions. As a result, a linear relationship between photocurrent and NO_2_^−^ concentration was established. Under optimized test conditions, the fabricated sensor demonstrated a wide linear range from 1 to 10 000 nM with a LOD of 0.48 nM (Fig. 10H).

## 4. Conclusion and outlook

Photoelectrochemical detection has emerged as a promising analytical method for tracing environmental pollutants due to its distinct advantages, including cost-effectiveness, rapid response, minimal background noise, and high sensitivity. Crucially, photoactive materials, which significantly influence the monitoring activity of PEC sensors, are key components of these sensing systems. In particular, porous materials have garnered considerable interest in the design and fabrication of photoelectrochemical sensing platforms due to their unique properties, such as a high surface area, tunable pore sizes, and an abundance of functional groups. In this review, we summarize recent advances in photoactive porous material-based photoelectrochemical sensors and their applications in monitoring environmental contaminants in water bodies based on a substantial body of research. We introduce and categorize typical porous materials into five groups: metal oxides, metal–organic frameworks, covalent organic frameworks, graphitic carbon nitride, and MXene. Additionally, we separately discuss their applications in detecting heavy metal ions, organic pollutants, and non-metal ion pollutants.

Most importantly, the structural effects of porous materials on the photoelectrochemical detection performance (sensitivity, detection limits, selectivity, and stability) of associated sensors have been discussed in detail in several representative works. Based on the comparison of numerous references, the structural effects of porous materials on PEC detection activity can be summarized into the following critical points: (1) enhancement of the light absorption ability through the multiple reflection of light in pores, improving the light harvesting capability; (2) acceleration of the photoinduced electron–hole pair separation and transfer through the unique porous structures with considerable small pores, shortening the separation and migration route and suppressing charge pair recombination; (3) improvement of the surface charge transfer rate, porous materials with the high surface area provide abundant active sites for chemical reactions and facilitate the detection of species capture. From the aforementioned advantages of porous materials for the construction of photoelectrochemical sensing platforms, we strongly confirm the remarkable potential of porous materials for environmental pollutant detection.

Despite excellent advances and rapid progress in the field of photoelectrochemical sensors based on porous materials for environmental toxic species assays, some fundamental issues and emerging challenges remain:

(1) Even though much effort has been devoted to the application of porous materials in PEC sensors for environmental hazard detection, only a small portion of the vast family of porous materials has been used to fabricate PEC sensing platforms. Meanwhile, some porous materials are only preliminary to PEC sensing applications for highly toxic species, such as photoactive COFs. Therefore, it still has huge potential for exploitation. Furthermore, relatively few porous materials have been used for heavy metal ion assays, only metal oxides and graphitic carbon nitride. Some toxic heavy metal ions, such as arsenic, have not been detected by porous material-based PEC sensors.

(2) Most studies of porous materials focus on the energy level structure and neglect the unique porous structures, *e.g.*, pore diameter and surface area. In addition, the effect of the porous structure on the photoinduced charge transfer and separation efficiency needs to be explored more. More importantly, although the photoelectric coordination of the detection mechanism based on the porous materials has been explained in several works, they only prefer to discuss the photocurrent signal generation mechanism in terms of the classical semiconductor theory and the consideration of the structural effects on the detection mechanism are somewhat insufficient. More attention should be paid to it for future studies, as porous structures favor trapping of probe species and an abundance of functional groups provides more active sites for the occurrence of relevant redox reactions. Moreover, the stability of PEC sensors based on porous materials requires more attention due to the fragile nature of porous structures and multiple assembly processes.

(3) The application of porous materials to PEC sensing is primarily at the laboratory stage. For practical applications, it still suffers from several shortcomings, such as relatively low reliability and reproducibility. Moreover, real samples may contain multiple environmental hazards, thus requiring multiplexing sensing capabilities for PEC sensors based on porous materials. In the field of environmental sample detection, integrating PEC detection methods with flexible electrodes, such as conductive polymers and carbon paper, is highly recommended. Flexible electrodes support the miniaturization and development of wearable and smart devices. PEC detection strategies should be combined with intelligent technologies to enable real-time and *in situ* detection, while still emphasizing the stability, convenience, and operability of integrated detection devices. Additionally, there are currently few commercial PEC sensing devices on the market, which hinders the development of PEC sensing methods. Therefore, future research should focus more on the fabrication of PEC-related equipment. With the help of smart technologies and the potential for commercial benefits, the current limitations of PEC sensing can be resolved.

## Data availability

The data that support the findings of this study are available from the corresponding author upon reasonable request.

## Author contributions

Shiben Liu, conceptualization, paper writing; Jinhua Zhan, analysis of article structure and error proofreading; Bin Cai, collecting data and providing financial support. Shiben Liu's writing, first draft preparation. Jinhua Zhan and Bin Cai's writing, review and proofreading.

## Conflicts of interest

The authors declare no conflict of interest.

## Supplementary Material
